# A Unique Dermal Dendritic Cell Subset That Skews the Immune Response toward Th2

**DOI:** 10.1371/journal.pone.0073270

**Published:** 2013-09-09

**Authors:** Ryuichi Murakami, Kaori Denda-Nagai, Shin-ichi Hashimoto, Shigenori Nagai, Masahira Hattori, Tatsuro Irimura

**Affiliations:** 1 Laboratory of Cancer Biology and Molecular Immunology, Graduate School of Pharmaceutical Sciences, the University of Tokyo, Tokyo, Japan; 2 Department of Computational Biology, Graduate School of Frontier Sciences, the University of Tokyo, Kashiwa, Japan; 3 Department of Microbiology and Immunology, Keio University School of Medicine, Tokyo, Japan; MRC National Institute for Medical Research, United Kingdom

## Abstract

Dendritic cell (DC) subsets in the skin and draining lymph nodes (LNs) are likely to elicit distinct immune response types. In skin and skin-draining LNs, a dermal DC subset expressing macrophage galactose-type C-type lectin 2 (MGL2/CD301b) was found distinct from migratory Langerhans cells (LCs) or CD103^+^ dermal DCs (dDCs). Lower expression levels of Th1-promoting and/or cross-presentation-related molecules were suggested by the transcriptome analysis and verified by the quantitative real-time PCR analysis in MGL2^+^ dDCs than in CD103^+^ dDCs. Transfer of MGL2^+^ dDCs but not CD103^+^ dDCs from FITC-sensitized mice induced a Th2-type immune response *in vivo* in a model of contact hypersensitivity. Targeting MGL2^+^ dDCs with a rat monoclonal antibody against MGL2 efficiently induced a humoral immune response with Th2-type properties, as determined by the antibody subclass. We propose that the properties of MGL2^+^ dDCs, are complementary to those of CD103^+^ dDCs and skew the immune response toward a Th2-type response.

## Introduction

Dendritic cells (DCs) recognize foreign materials and play a central role in the initiation of a variety of immune responses [1], [2]. However, it is not yet fully understood how DCs determine the type, strength, duration, localization, memory, and other aspects of the immune response. Interestingly, DCs residing in or migrating into various organs seem to be distinct and potentially be classified into subsets according to surface marker molecules and functions [3], [4]. These DC subsets have been suggested to have distinct roles in the initiation of different types of immune responses [3], [5], [6].

At least several DC subsets are known to reside in skin and skin-draining lymph nodes (LNs) [7], [8], [9], [10]. For example, Langerhans cells (LCs) constitute one of the skin DC subsets, and Langerin was thought to be a specific marker of LCs for a period of time [11]. Recently, however, a new DC subset expressing Langerin, the CD103^+^ dermal dendritic cells (dDCs), was found in the skin immune system, and it was shown to be distinct from migratory LCs based on the expression of distinct surface markers and its unique function [12], [13], [14], [15]. In addition, it was shown that LCs and CD103^+^ dDCs promote opposite T cell response types, Th17- and Th1- type, respectively, suggesting that skin DC subsets are specialized to induce distinct immune responses [16], [17].

Contact hypersensitivity (CHS) is T cell-mediated immunity with the characteristics of delayed-type hypersensitivity [18], [19]. CHS is experimentally induced by painting haptens diluted in adjuvants onto the skin. Two important phases are involved in CHS reactions: the sensitization phase and the elicitation phase [20]. Classically, LCs were considered to be the main antigen presenting cells (APCs) in the sensitization phase of CHS [18], [21], [22]. However, recent studies using new technologies to deplete LCs have provided confusing information because the depletion of LCs has been shown to promote [23], to have no effect on [12], [24], and to suppress CHS [25], [26], [27], [28]. Furthermore, a new dDC subset, the CD103^+^ dDCs, was reported to be involved in the initiation of CHS responses [12], [25]. Finally, it was recently shown that antigen presentation by CD103^+^ dDCs alone did not appear to represent the main pathway involved in sensitization for CHS [29]. Therefore, which skin DC subsets play the dominant role in CHS remains controversial.

We propose in the present report that dDCs expressing macrophage galactose (Gal)-type C-type lectin 2 (MGL2/CD301b) comprise a unique subset. MGL2 is a type II transmembrane lectin containing a single carbohydrate recognition domain that interacts with Gal and *N*-acethylgalactosamine (GalNAc) as monosaccharides and binds strongly to oligosaccharides with terminal GalNAc residues. Although a single gene encodes human MGL/CD301 [30], mice have two genes: *Mgl1* [31] and *Mgl2* [32]. We recently examined the distribution of cells expressing MGL2 and concluded that MGL2 was expressed on a portion of conventional DCs [33]. In the skin, MGL2 was expressed on a portion of dDCs that were sufficient to initiate CHS *in vivo* [34]. However, it was not clear whether MGL2^+^ cells overlapped with or were distinct from migratory LCs and CD103^+^ dDCs.

In the present report, MGL2^+^ dDCs are found to comprise a unique subset that is distinct from migratory LCs, CD103^+^ dDCs, CD8á^+^ DCs, or plasmacytoid DCs (pDCs) in the skin immune system. The quantitative real-time PCR analysis demonstrated that the expression levels of Th1-promoting and/or cross-presentation-related molecules were lower in MGL2^+^ dDCs than in CD103^+^ dDCs. To assess the role of MGL2^+^ dDCs in CHS *in vivo*, MGL2^+^ dDCs bearing a hapten, fluorescence isothiocyanate (FITC), and CD103^+^ dDCs bearing the same hapten were sorted and injected into naïve mice; then the subsequent immune responses were examined. Both MGL2^+^ dDCs and CD103^+^ dDCs initiated CHS sensitization to a similar extent as revealed by the thickening of the ears. However, MGL2^+^ dDCs but not CD103^+^ dDCs were shown to induce a Th2-type immune response *in vivo*, in the profiles of cytokines and the antibody subclass, in FITC-induced CHS. Furthermore, Th2 skewed humoral immune responses were observed when an antigen was targeted to MGL2^+^ dDCs *in vivo*. These results revealed for the first time that MGL2^+^ dDCs represent a unique dDC subset for the Th2-type immune response.

## Materials and Methods

### Mice

Five-week-old specific pathogen-free (SPF) BALB/c or C57BL/6 mice of both sexes were purchased from CLEA Japan, Inc. (Tokyo, Japan). Purchased mice, mice transgenic for the OVA_323–339_-specific and I-A^d^-restricted DO11.10 TCR-αβ on a *Rag2* ^–/–^ (BALB/c) background and *Mgl2* ^–/–^ mice (BALB/c background) were fed and housed under SPF conditions. All experiments were approved by the Bioscience Committee of the Graduate School of Pharmaceutical Sciences of the University of Tokyo and performed according to the guidelines of the Bioscience Committee of the University of Tokyo.

### Sensitization for CHS

BALB/c mice were painted with 200 µl of 0.5 % (w/v) FITC (Dojindo Laboratories, Kumamoto, Japan) in AD, a 1:1 mixture of acetone (Wako Pure Chemical, Osaka, Japan) and dibutylphthalate (Wako), on shaved arms and abdomen.

### Preparation of skin cells

Shaved skin was obtained from mice under a naïve state. After removing the adipose tissue, skin samples were minced and digested with 6 mg/ml collagenase (Wako) and 0.1 mg/ml DNaseI (Roche, Basel, Switzerland) in RPMI1640 medium supplemented with 10% fetal calf serum (FCS) for 75 min at 37°C. Digested cells were transferred to new tubes through iron mesh, centrifuged, treated with 10 mM EDTA in PBS for 5 min, washed two times with PBS, and suspended in PBS containing 0.1% BSA and 0.1% sodium azide (FCM buffer).

### Preparation of LN cells

Brachial, axillary and inguinal LNs were obtained from BALB/c mice under a naïve state or 1 day after sensitization for CHS. Draining brachial and axillary LNs were obtained from BALB/c mice 1 day after CHS elicitation when mice were sensitized with FITC^+^ dDCs by footpad injection. LNs were minced and digested with 1 mg/ml collagenase from Clostridium histolyticum (Sigma) in RPMI1640 medium supplemented with 10% FCS for 25 min at 37°C. Digested cells were transferred to a new tube through nylon mesh and washed with PBS containing 0.5% BSA and 2 mM EDTA.

### Antibodies

FITC-conjugated anti-mouse IA/IE (monoclonal antibody (mAb) M5/114.15.2), anti-CD11c (mAb N418), anti-CD4 (mAb RM4-5), phycoerythrin (PE)-conjugated anti-mouse CD103 (mAb 2E7), anti-mouse Ep-CAM (mAb G8.8), anti-mouse B220 (mAb RA3-6B2), anti-mouse CD4 (mAb RM4-5), anti-mouse IFN-γ (mAb XMG1.2), anti-mouse IL-17A (mAb TC11-18H10.1), biotin-conjugated anti-mouse CD4 (mAb GK1.5), anti-mouse CD8α (mAb 53-6.7), anti-mouse CD80 (mAb 16-10A1), anti-mouse CD86 (mAb GL-1), anti-mouse B7-H1 (mAb 10F.9G2), anti-mouse B7-H2 (mAb HK5.3), anti-mouse B7-DC (mAb TY25), anti-mouse IL-12/IL-23p40 (mAb C17.8), PE-Cy7-conjugated anti-mouse CD3ε (mAb 145-2C11), Alexa Fluor 488-conjugated anti-mouse Foxp3 (mAb 150D), and allophycocyanin (APC)-conjugated anti-mouse IFN-γ (mAb XMG1.2) were purchased from BioLegend (San Diego, CA). Biotin-conjugated anti-mouse TCRVâ8 (JR2) purchased from BD Biosciences (Oxford, U.K.). PE-conjugated anti-mouse Langerin (mAb eBioL31), anti-mouse IL-4 (mAb 11B11), and biotin-conjugated anti-mouse CD40 (mAb HM40-3) were purchased from eBioscience (San Diego, CA). PE-conjugated anti-mouse mPDCA-1 (mAb JF05-1C2.4.1) was purchased from Miltenyi Biotec (Bergisch Gladbach, Germany). Biotinylated-, APC- or Hilyte647-conjugated anti-mouse MGL2 (mAb URA-1) was prepared using purified antibodies from the hybridoma culture supernatant [33].

### Flow cytometry analysis

Cells isolated from mouse tissues and primary cultured cells were incubated with anti-mouse CD16/CD32 mAb (1/100 dilution of ammonium sulfate-precipitated hybridoma culture supernatant; the 2.4G2 hybridoma was purchased from ATCC) to reduce non-specific binding 5 min before the addition of the first antibodies. Cells were then incubated with biotin-, FITC-, PE-, PE-Cy7-, HiLyte647- and/or APC-conjugated antibodies for 30 min. Biotin-conjugated antibodies were visualized with PE-Cy7- or APC-labeled streptavidin (SAv) (BioLegend). To exclude dead cells, except for intracellularly stained cells, all cells were resuspended in FCM buffer containing 7-amino-actinomycin D (7-AAD) (eBioscience). To stain intracellular molecules, cells were fixed and permeabilized with BD Cytofix/Cytoperm (BD Biosciences, Franklin Lakes, NJ) according to the manufacturer's protocol. All procedures were performed on ice. Antibodies and reagents were diluted in FCM buffer. The cells were rinsed once with FCM buffer at the end of each incubation period. Samples were analyzed on a FACS Aria cell sorter (BD). Data were analyzed using FlowJo software (Tree Star, Ashland, OR).

### Sorting of MGL2^+^ dDCs and CD103^+^ dDCs

After the preparation of LN cells, CD11c^+^ cells were isolated by an autoMACS Separator (Miltenyi) according to the manufacturer's protocol. After MACS, cells were stained with mAbs and suspended in sterilized 3% FCS-PBS. MHCII^high^MGL2^+^ dDCs and MHCII^high^CD103^+^ dDCs were sorted from mice under a naïve state by a FACS Aria cell sorter. FITC^+^MGL2^+^ dDCs and FITC^+^CD103^+^ dDCs were sorted from BALB/c mice 1 day after sensitization for CHS by a FACS Aria cell sorter. After sorting, the purity of each dDC subset was at least 90% or more.

### Encyclopedic transcriptome analysis

MHCII^high^MGL2^+^ dDCs, MHCII^high^CD103^+^ dDCs, FITC^+^MGL2^+^ dDCs or FITC^+^CD103^+^ dDCs were obtained from forty mice and pooled. Total RNA was obtained using an RNeasy Mini Kit (QIAGEN, Valencia, CA). Individual SAGE libraries from the dDC samples were constructed with 1 µg RNA using the SOLiD SAGE™ kit from Life Technologies (Beverly, MA) according to the manufacturer's protocol. The constructed tags were sequenced by a SOLiD4 sequencer (Life Technologies Japan). The samples were mapped to Refseq using the v1.1 SOLiD™ SAGE™ Analysis software method, which used the 25_1 mapping parameter. After mapping, the number of genes for each dDC subset was normalized by setting the number of total genes to 3000000. After normalization, the number of genes was rounded up to the nearest integral number. Encyclopedic transcriptome analysis was performed, and the fold differences in the relative expression levels of indicated genes (*Cxcl2, Cxcl3, Ccl1, Il12b, Xcr1, Naip2, Clec4n, Clec9a* and *Tlr3*) among the dDC subsets (MHCII^hi^MGL2^+^ dDCs, MHCII^hi^CD103^+^ dDCs, FITC^+^MGL2^+^ dDCs and FITC^+^CD103^+^ dDCs). Differential expressions suggested by the encyclopedic transcriptome analysis were verified by the quantitative real-time PCR more than two times.

### Accession number

SAGE tags have been deposited in the NCBI Short Read Archive under the project accession, DRA000852.

### Quantitative real-time PCR

Draining brachial and axillary LN cells were obtained 1 day after CHS elicitation from mice sensitized with FITC^+^MGL2^+^ dDCs, FITC^+^CD103^+^ dDCs or non-sensitized mice. MHCII^high^MGL2^+^ dDCs, MHCII^high^CD103^+^ dDCs, FITC^+^MGL2^+^ dDCs or FITC^+^CD103^+^ dDCs were obtained as shown above. Total RNA was obtained from these cells with an RNeasy mini kit or RNeasy micro kit (QIAGEN). RNA (0.05–0.4 µg) was reverse-transcribed into cDNA using Superscript III (Invitrogen). All procedures were performed according to the manufacturers' instructions. The quantitative real-time PCR was performed on a StepOnePlus Real-time PCR System (Applied Biosystems, Foster City, CA) using Fast SYBR Green master mix (Applied Biosystems) or KAPA SYBR FAST ABI Prism (Kapa Biosystems,Woburn, MA). Primers are listed in Table S3.

### Intracellular analysis of IL-12_p40_ on skin-derived DCs in LNs

LN cells (4.0×10^6^ cells) obtained from BALB/c mice under a naïve state or mice 1 day after sensitization for CHS were cultured for 2 hours in the presence of 10 µg/ml brefeldin A (Sigma) on 24-well plates. After incubation, intracellular IL-12_p40_ in skin-derived DCs was analyzed by a FACS Aria cell sorter.

### Cytokine production from MGL2^+^ dDCs and CD103^+^ dDCs

MHCII^high^MGL2^+^ dDCs, MHCII^high^CD103^+^ dDCs, FITC^+^MGL2^+^ dDCs or FITC^+^CD103^+^ dDCs were obtained as shown above. dDCs (2.0×10^4^ cells) were cultured in the presence of 10 µg/ml anti-CD40 agonistic antibody (FGK-45) for 15 hours on 96-well round-bottom plates. Cytokine concentrations in culture supernatant were measured using the Bio-Plex Suspension Array System (Bio-Rad Laboratories, Hercules, CA).

### Adoptive transfer and CHS elicitation

Sorted FITC^+^MGL2^+^ dDCs and FITC^+^CD103^+^ dDCs (2.0×10^4^ cells) were resuspended in 10 µl of Hanks' balanced salt solution (HBSS) and injected into the footpads of BALB/c mice under a naïve state. Six days later, the recipient mice were painted with 20 µl of 0.5% FITC/AD on the skin of the dorsal right ear. The left ear was treated with the vehicle alone as a control. As negative controls for sensitization, BALB/c mice that were injected with HBSS 6 days prior to elicitation (non-sensitized mice) were used. FITC-specific ear swelling was defined as follows: (right ear thickness at 24 h after elicitation – right ear thickness before elicitation) – (left ear thickness at 24 h after elicitation – left ear thickness before elicitation). For intracellular cytokine staining and FITC-specific antibody ELISA, draining brachial and axillary LN cells and sera were obtained 1 day after elicitation from mice sensitized with FITC^+^MGL2^+^ dDCs, FITC^+^CD103^+^ dDCs or non-sensitized mice.

### Intracellular analysis of cytokines in T cells in LNs after CHS elicitation

LN cells (4.0×10^6^ cells) obtained 1 day after CHS elicitation from mice sensitized with FITC^+^MGL2^+^ dDCs, FITC^+^CD103^+^ dDCs or non-sensitized mice were cultured for 5 hours in the presence of 20 ng/ml phorbol 12-myristate 13-acetate (PMA) (Sigma), 1 µM ionomycin calcium salt from *Streptomyces conglobatus* (ionomycin) (Calbiochem) and 10 µg/ml brefeldin A in a 24-well plate. After culturing, intracellular cytokines in CD4^+^ T cells were analyzed by a FACS Aria cell sorter.

### FITC-specific antibody ELISA

Ninety-six-well ELISA microplates (Greiner, Monroe, NC) were coated with fluorescein-conjugated BSA (FITC-BSA; 4 µg/ml: Invitrogen) or BSA (4 µg/ml: Calbiochem, Darmstadt, Germany) and incubated at 4°C overnight. The plates were washed with 0.05% Tween-20-PBS and blocked with 10% FCS-PBS for 1 hour at room temperature. Sera from mice were diluted in 10% FCS-PBS and incubated for 2 hours at room temperature. Goat anti-mouse IgG1 and IgG2a and human ads-HRP (Beckman Coulter, Fullerton, CA) were added, and the plates were incubated for 1 hour at room temperature. The substrate, 3,3,5,5-tetramethylbenzidine (Sigma: 0.1 mg/ml) diluted in 0.05 M citrate-phosphate buffer (pH5) containing hydrogen peroxide (Wako), was added, and the reaction continued for 30 minutes. The reaction was terminated by the addition of 2 N sulfuric acid, and the OD 450 nm/570 nm was measured by ARVO X5 (PerkinElmer Japan, Kanagawa, Japan).

### OVA-specific CD4^+^ T cell differentiation assays

CD4^+^ T cells were purified from the spleens of DO11.10*rag2* ^–/–^ mice by MACS. FITC^+^MGL2^+^ dDCs and FITC^+^CD103^+^ dDCs or MHCII^high^MGL2^+^ dDCs and MHCII^high^CD103^+^ dDCs were obtained as shown above. dDCs (1.0×10^4^ cells) were cultured in the presence or absence of 2 µg/ml OVA_323–339_ peptide (Genscript) for 2 hours and added with CD4^+^ T cells (1.0×10^5^ cells). The co-culture was continued for 3 or 5 days in 96-well round-bottomed plates. After the culture supernatants were obtained, the cells were cultured in the presence of 20 ng/ml PMA, 1 µM ionomycin, and 10 µg/ml Brefeldin A for 5 hours. Cytokine concentrations in culture supernatant were measured using the Bio-Plex Suspension Array System. Intracellular cytokines in T cells were measured by a FACS Aria cell sorter.

### Targeting MGL2^+^ dDCs and analysis of subsequent immune responses

Anti-MGL2 mAbs or isotype control Rat IgG2a (5 µg) together with LPS (1 µg) were injected into the footpad of *Mgl2* ^+/+^ or *Mgl2* ^–/–^ mice (BALB/c background). One day after the injection of biotinylated anti-MGL2 mAbs, cells from draining LNs stained with anti-CD11c, anti-MGL2, and intracellular PE-SAv were analyzed by a FACS Aria cell sorter. One week after injection, sera were obtained. Anti-Rat IgG2a antibodies in sera were determined by ELISA using HRP-goat anti-mouse IgGAM (Zymed), IgM, IgG1, IgG2a, IgG2b, and IgG3 (Beckman Coulter).

### Statistical analysis

Data were compared with a two-sided student's *t* test and presented as the mean ± standard deviation (SD). When *p* values were less than 0.05, the difference was considered significant.

## Results

### MGL2^+^ dDCs are distinct from migratory LCs and CD103^+^ dDCs in skin under a naïve state

**Figure 1:**
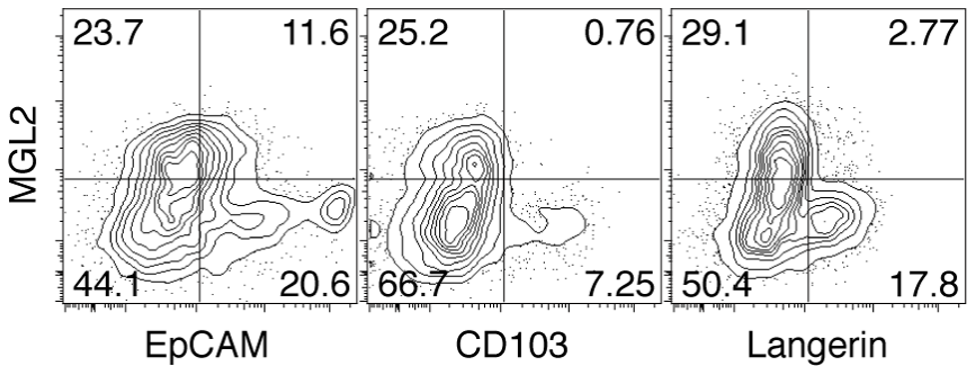
Characterization of MGL2^+^ dDCs in skin. Flow cytometry analysis of skin cell suspensions for DC markers. MHCII^+^ cells were analyzed for the expression of MGL2, EpCAM, CD103, and Langerin. The experiments were independently performed three times.

The expression of MGL2 is unique to dermal DCs and is not observed with epidermal cells such as epidermal LCs [34]. However, whether MGL2 is also expressed on migratory LCs, CD103^+^ dDCs, or other DC subsets distinct from these populations has not been previously studied. We analyzed skin cells from mice under a naïve state by flow cytometry and found that almost all MHCII^+^ cells were CD11c^+^ and thus DCs (data not shown). MGL2^+^ cells belonged to this population and were shown to represent EpCAM^low^CD103^–^Langerin^–^ cells (**Fig. 1**). Previous reports showed that LCs had characteristics of EpCAM^high^CD103^–^Langerin^+^ cells [12], [15], [35], and CD103^+^ dDCs had characteristics of EpCAM^low^CD103^+^Langerin^+^ cells [12], [15], [35]. Therefore, MGL2^+^ dDCs should be distinct from migratory LCs and from CD103^+^ dDCs in skin.

### MGL2^+^ dDCs are distinct from LCs or CD103^+^ dDCs in skin-draining LNs under a naïve state and after sensitization for CHS

**Figure 2:**
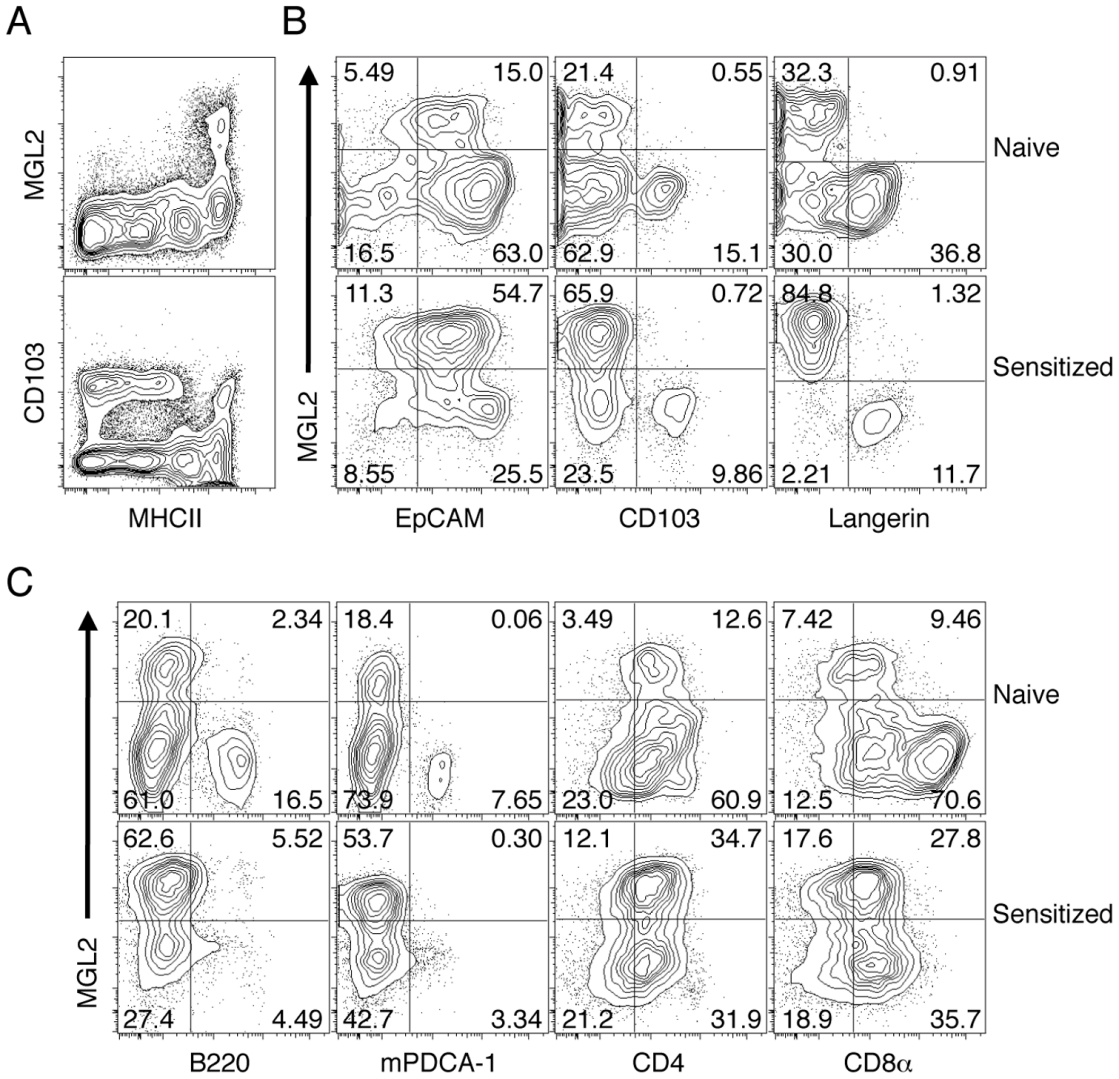
Characterization of MGL2^+^ dDCs in skin-draining LNs. Flow cytometry analysis of cells from skin-draining LNs for DC markers. (A) LN cells were analyzed for the expression of MGL2 or CD103 and MHCII. (B) MHCII^high^ cells from naïve mice are shown as “Naive” and FITC^+^ cells from sensitized mice (1 day after FITC painting) are shown as “Sensitized.” MHCII^high^ cells or FITC^+^ cells were analyzed for the expression of MGL2, EpCAM, CD103, or Langerin. (C) CD11c^+^ cells from naïve mice, shown as “Naïve,” and FITC^+^ cells from sensitized mice, shown as “Sensitized,” were analyzed for the expression of MGL2, B220, mPDCA-1, CD4, or CD8á. (A–C) The experiments were independently performed three times.

In skin-draining LNs under a naïve state, MGL2^+^ dDCs are CD11c^+^CD11b^high^MHCII^high^CD86^+^CD40^high^F4/80^+^B220^−^Ly6G^−^CD8á^low^ and represent a uniform subset [34]. However, MGL2^+^ dDCs in LNs might have contained cells derived from migratory LCs or CD103^+^ dDCs. Alternatively, MGL2^+^ dDCs were distinct from these well-known subsets in skin-draining LNs. To clarify this point, we analyzed LN cells from mice under a naïve state and 1 day after sensitization for CHS with FITC for the distribution of MGL2 and other markers on skin-derived DCs by flow cytometry. All MHCII^high^ cells were confirmed to be CD11c^+^ under a naïve state and 1 day after percutaneous administration of FITC (data not shown), indicating that MHCII^high^ cells in LNs were DCs derived from the skin [35]. We also confirmed that all cells containing FITC in skin-draining LNs 1 day after sensitization were MHCII^high^ (data not shown). These results indicated that FITC was mainly processed by skin-derived DCs but not by resident DCs in LNs. In addition, MGL2 but not CD103 was specifically expressed on cells in the MHCII^high^ compartment in the flow cytometry charts of cells from skin-draining LNs under a naïve state (**Fig. 2A**). These findings also confirmed that MGL2 was specifically expressed on skin-derived DCs in LNs. The results shown in **Fig. 2B** revealed that MHCII^high^MGL2^+^ cells in skin-draining LNs under a naïve state and FITC^+^MGL2^+^ cells 1 day after sensitization for CHS with FITC were EpCAM^int^CD103^–^Langerin^–^. Because the LCs that migrate into skin-draining LNs are EpCAM^high^CD103^–^Langerin^+^ [12], [15], [35], and CD103^+^ dDCs in LNs are EpCAM^int^CD103^+^Langerin^+^ [12], [15], [35], our results clearly indicate that MGL2^+^ dDCs are distinct from migratory LCs or CD103^+^ dDCs in skin-draining LNs both under naïve and sensitized conditions. Based on the notion that skin-draining LNs contain LN-resident CD8á^+^ DCs [35], [36], [37] and pDCs [37], [38], we assessed whether MGL2 was present on these DC subsets in naïve and sensitized skin-draining LNs. In both cases, MGL2^+^ cells were CD4^low^CD8á^low^B220^–^mPDCA-1^–^ (**Fig. 2C**). According to previous reports, the majority of LN-resident DCs highly express CD8á [37], and pDCs express B220 [38] and mPDCA-1 [39], whereas MGL2 was not detected on CD8á^+^ DCs or on pDCs in skin-draining LNs. Thus, we concluded that there is no overlap between these populations and MGL2^+^ cells. We also confirmed that CD8á^+^ DCs or pDCs did not incorporate FITC hapten during experimental CHS as previously shown [24].

To explore the differences between MGL2^+^ dDCs and the other skin-derived DC subsets, we compared the expression of co-stimulatory molecules on MGL2^+^ dDCs, MGL2^–^CD103^–^ skin-derived DCs (DN DCs), and CD103^+^ dDCs obtained from skin-draining LNs under a naïve state and after sensitization for CHS with FITC. The profiles of CD40, CD80, CD86, B7-H1, and B7-H2 were not different among these three skin-derived DC subsets, regardless of the sensitization for CHS (**Figs. S1A and S1B**). Interestingly, the level of B7-DC on MGL2^+^ dDCs was higher than for DN DCs or CD103^+^ dDCs (**Figs. S1A and S1B**). The levels of CD40, CD80, CD86, B7-H1, and B7-DC on MGL2^+^ dDCs, DN DCs, and CD103^+^ dDCs were elevated one day after sensitization for CHS, but B7-H2 was not elevated (**Figs. S1A and S1B**), indicating that all skin-derived DC subsets underwent maturation to a similar extent after sensitization for CHS.

### Encyclopedic transcriptome analysis and quantitative real-time PCR for MGL2^+^ dDCs and CD103^+^ dDCs

**Figure 3:**
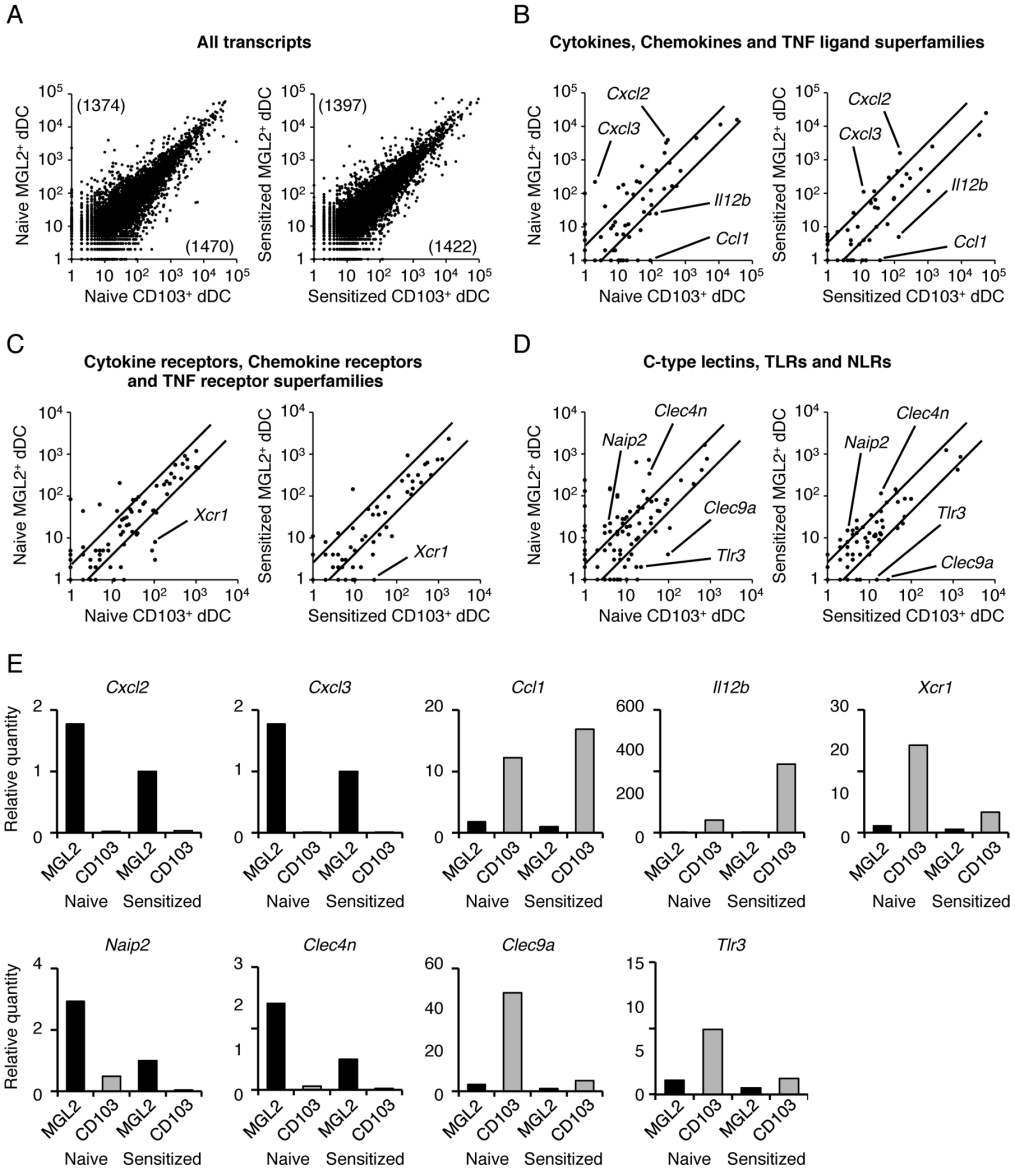
Encyclopedic transcriptome analysis of MGL2^+^ dDCs and CD103^+^ dDCs and results of quantitative real-time PCR for the transcripts suggested by the transcriptome analysis to have differential levels. (A–D) Reciprocal two-dimensional plots of the results of transcriptome analysis among MHCII^high^MGL2^+^ cells, MHCII^high^CD103^+^ cells, FITC^+^MGL2^+^ cells and FITC^+^CD103^+^ cells. In these panels, MHCII^high^MGL2^+^ dDCs are indicated as “Naïve MGL2^+^ dDC,” MHCII^high^CD103^+^ dDCs are indicated as “Naïve CD103^+^ dDC,” FITC^+^MGL2^+^ dDCs are indicated as “Sensitized MGL2^+^ dDC,” and FITC^+^CD103^+^ dDCs are indicated as “Sensitized CD103^+^ dDC.” (A) All transcripts of cells from naïve mice and cells from sensitized mice are shown. The numbers in the parentheses indicate the number of transcripts expressed 3 times greater than the other dDC subset. (B–D) The numbers of transcripts in selected categories are plotted. In the comparisons, the names of the transcripts are indicated when they fit the following criteria: (1) the number is greater than 15 in MGL2^+^ dDCs or in CD103^+^ dDCs, and (2) the difference in the number is 5-fold or greater both before and after sensitization. The diagonal lines represent the border for 3-fold differences. (B) Transcripts of cytokines, chemokines and TNF ligand superfamily members are shown. (C) Transcripts of cytokine receptors, chemokine receptors, and TNF receptor superfamily members are shown. (D) Transcripts of C-type lectins, TLRs and NLRs are shown. (E) The quantitative real-time PCR analysis of the expression of indicated genes (*Cxcl2, Cxcl3, Ccl1, Il12b, Xcr1, Naip2, Clec4n, Clec9a* and *Tlr3*). They were chosen from the categories indicated above (B), (C), and (D) and the differences between MGL2^+^ dDCs and CD103^+^ dDCs based on the transcriptome analysis, appeared to be significant under both untreated and sensitized conditions (Figs. 3B–D). (A–E) Transcriptome analysis was performed once. The quantitative real-time PCR analysis was independently performed more than two times.

MGL2^+^ dDCs and CD103^+^ dDCs before and after sensitization for CHS were subjected to encyclopedic transcriptome analysis. Among the 18521 transcripts that were identified, approximately 1400 transcripts were estimated to be present in numbers that were more than three times greater in MGL2^+^ dDCs than in CD103^+^ dDCs under both naïve and sensitized conditions (**Fig. 3A**). Additionally, under both naïve and sensitized conditions, approximately 1400 transcripts were estimated in numbers that were more than three times greater in CD103^+^ dDCs than in MGL2^+^ dDCs (**Fig. 3A**). These results suggested that transcript expression profiles were distinct for MGL2^+^ dDCs and CD103^+^ dDCs under both naïve and sensitized conditions, despite the fact that both MGL2^+^ dDCs and CD103^+^ dDCs are skin-derived dDCs.

We focused on the profiles of cytokines, chemokines, TNF ligand superfamily members. TLRs, NLRs, and C-type lectins, which were thought to be involved in the regulation of DC functions (**Figs. 3B–3D** **, and Table S1**). Fold differences in the relative expression levels of the indicated genes (*Cxcl2, Cxcl3, Ccl1, Il12b, Xcr1, Naip2, Clec4n, Clec9a* and *Tlr3*) among the dDC subsets were determined by the quantitative real-time PCR (**Fig. 3E**).

From the results, it was found that MGL2^+^ dDCs expressed *Cxcl2* and *Cxcl3,* both of which were known to attract neutrophils, at higher levels than CD103^+^ dDCs [40], [41], [42]. MGL2^+^ dDCs expressed *Il12b,* which makes up a subunit of IL-12_p70_ that is critical for Th1 cell differentiation [43], [44] , at lower levels than CD103^+^ dDCs did. The level of *Ccl1,* which is known to attract various types of immune cells, in MGL2^+^ DCs was also lower than in CD103^+^ dDCs under naïve or sensitized conditions [45], [46], [47] (**Figs. 3B and 3E** **, and Table S1**). Among chemokine receptors and TNF receptor superfamily members, the levels of *Xcr1,* which is involved in cross-presentation [48], were lower in MGL2^+^ dDCs than in CD103^+^ dDCs (**Figs. 3C and 3E** **, and Table S1**). Among the variety of TLRs, NLRs, and C-type lectins that were examined, the levels of *Naip2*, which is known to form the NAIP-NALC4 inflammasome [49], [50], and the levels of *Clec4n*, which induces signal transduction and subsequent cytokine production after recognition of α-mannan derived from *C. albicans* [51], [52], were higher in MGL2^+^ dDCs than in CD103^+^ dDCs. Conversely, the levels of *Tlr3* and *Clec9a*, which are involved in cross-presentation [53], [54], [55], [56], [57], [58], [59], [60], [61], were lower in MGL2^+^ dDCs than in CD103^+^ dDCs (**Figs. 3D and 3E** **, and Table S1**). The levels of *Il18,* which is involved in Th2 cell differentiation in the absence of IL-12 [62], were slightly higher in MGL2^+^ dDCs than in CD103^+^ dDCs, both under naïve and sensitized conditions (**Table S1 and S2**). However, the levels of other Th2-promoting molecules such as *Il10* [63], *Tnfsf4* [64], [65], *jag1* [66], [67], *jag2* [66] and *Crlf2* (TSLP receptor) [68] were very similar between MGL2^+^ dDCs and CD103^+^ dDCs both under naïve and sensitized conditions (**Table S1 and Table S2**). Judging from these results, the levels of molecules promoting a Th1-type immune response and molecules potentially involved in cross-presentation were suggested to be expressed at much lower levels in MGL2^+^ dDCs than in CD103^+^ dDCs, whereas the levels of molecules promoting a Th2-type immune response were suggested to be almost similar in MGL2^+^ dDCs compared to CD103^+^ dDCs.

### The production levels of IL-12 by MGL2^+^ dDCs are low even after sensitization

**Figure 4:**
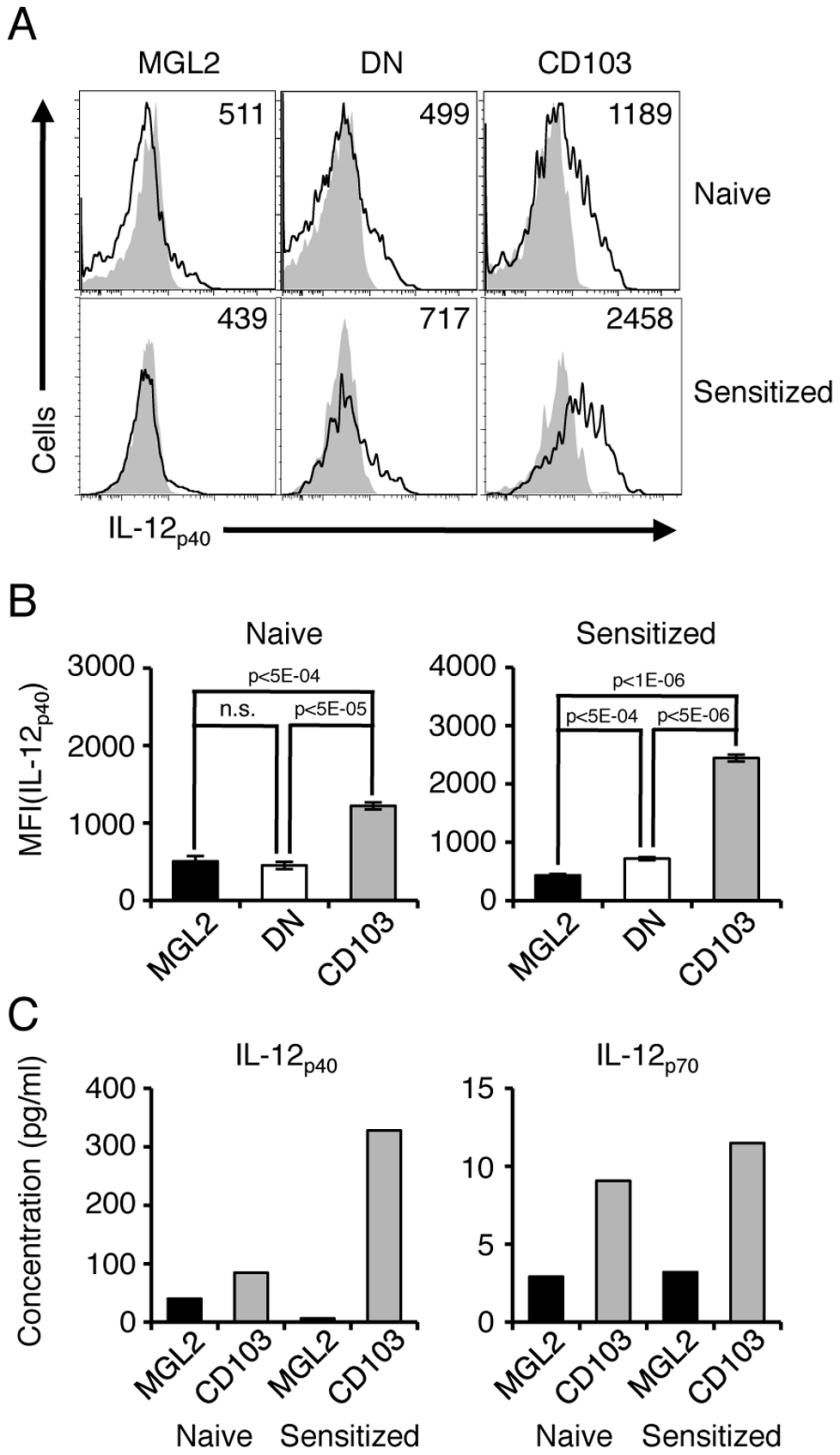
Paucity in IL-12 production by MGL2^+^ dDCs. (A) Flow cytometry analysis of intracellular IL-12_p40_ in MGL2^+^ dDCs, MGL2^–^CD103^–^ skin-derived DCs, and CD103^+^ dDCs in skin-draining LNs under naïve or sensitized conditions. MGL2^+^ dDCs are shown as “MGL2,” MGL2^–^CD103^–^ DCs are shown as “DN” and CD103^+^ dDCs are shown as “CD103.” Cells from naïve mice are shown as “Naïve,” and cells from sensitized mice are shown as “Sensitized.” Areas shaded in gray indicate the staining pattern with an isotype control antibody. The numbers represent the mean fluorescence intensity (MFI) of each skin-derived DC subset. (B) The MFI of IL-12_p40_ for each skin-derived DC subset is shown in panel A in bar graphs. Data are shown as the mean ± SD of three biological replicates (n = 3); n.s. indicates that the difference is not statistically significant. (A–B) The experiments were independently performed three times. (C) Concentrations of IL-12_p40_ and IL-12_p70_ in culture supernatant of MHCII^high^MGL2^+^ dDCs, MHCII^high^CD103^+^ dDCs, FITC^+^MGL2^+^ dDCs, and FITC^+^CD103^+^ dDCs were measured by the Bio-Plex Suspension Array System and are shown in the panels as “Naive MGL2,” “Naïve CD103,” “Sensitized MGL2,” and “Sensitized CD103,” respectively. The experiments were independently performed two times.

As shown above, the levels of *Il12b* transcript in MGL2^+^ dDCs were lower than in CD103^+^ dDCs, both under naïve and sensitized conditions. To quantify the corresponding protein, skin-draining LN cells were obtained from naïve and sensitized mice, and intracellular IL-12_p40_ on skin-derived DC subsets was measured by flow cytometry after treatment with brefeldin A. The level of intracellular IL-12_p40_ in MGL2^+^ dDCs was almost the same as that in DN DCs and was significantly lower than CD103^+^ dDCs under naïve conditions (**Figs. 4A and 4B**). One day after sensitization for CHS, intracellular IL-12_p40_ in MGL2^+^ dDCs was significantly lower than that found in DN DCs or CD103^+^ dDCs (**Figs. 4** **A and 4B**). The levels of secreted IL-12_p40_ and IL-12_p70_ by MGL2^+^ dDCs and CD103^+^ dDCs were also tested after sorting these cells from mice under naïve and sensitized conditions. The cells were cultured for 15 hours in the presence of an agonistic anti-CD40 antibody, and the concentrations of IL-12_p40_ and IL-12_p70_ in culture supernatants were examined. Concentrations of IL-12_p40_ and IL-12_p70_ in the culture supernatant of MGL2^+^ dDCs were much lower than those of CD103^+^ dDCs, both under naïve and sensitized conditions (**Fig. 4** **C**). These results indicated that MGL2^+^ dDCs produced a smaller amount of IL-12, a key cytokine for the Th1-type immune response, than CD103^+^ dDCs [43], [44] under both naïve and sensitized conditions.

### MGL2^+^ dDCs induce a Th2-type immune response *in vivo* in a model of contact hypersensitivity

**Figure 5:**
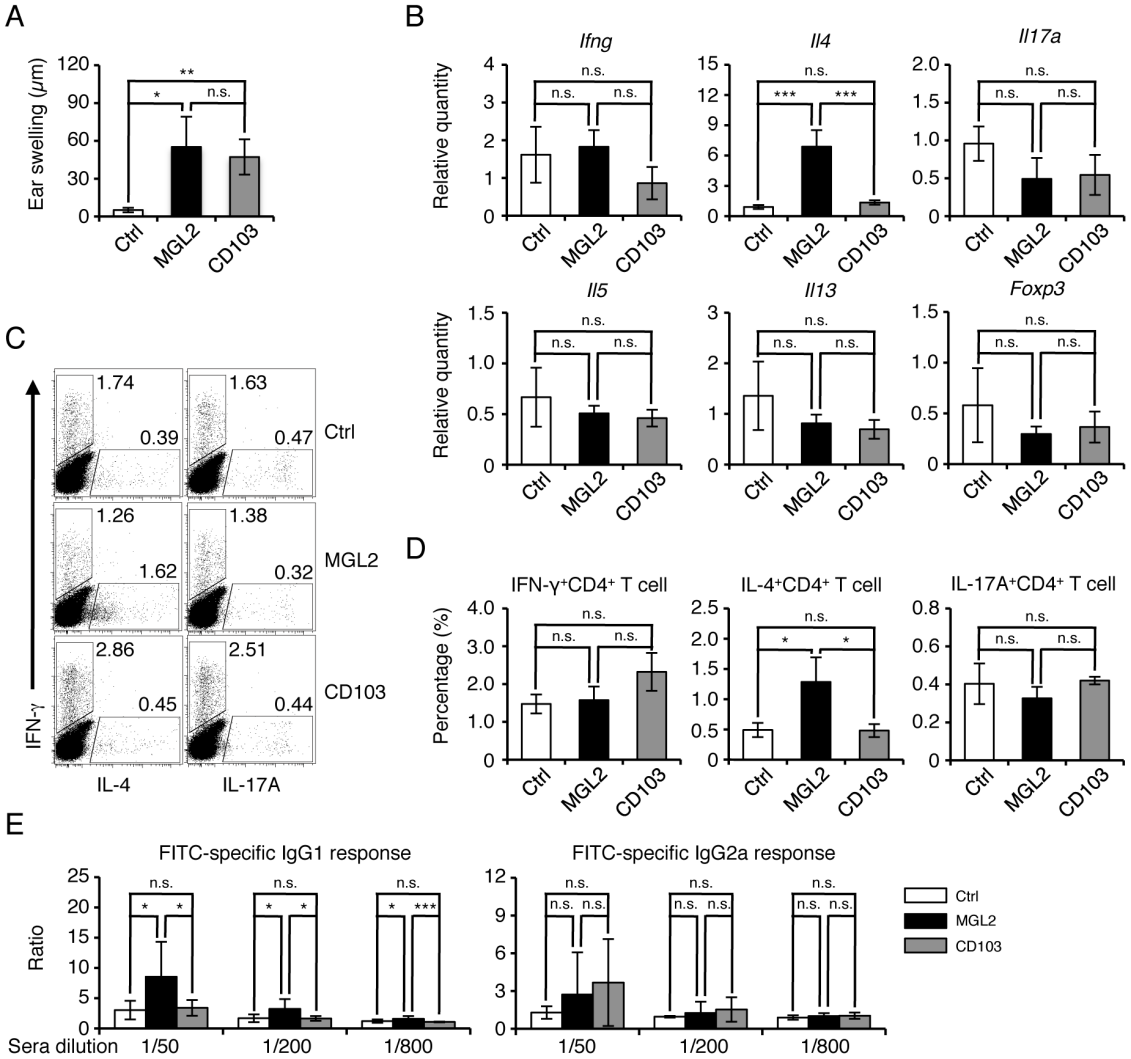
Immune response in mice after transfers of MGL2^+^ dDCs *in vivo*. In the following panels, data obtained from untreated mice, mice that received FITC^+^MGL2^+^ cells, and mice that received FITC^+^CD103^+^ cells are indicated as “Ctrl,” “MGL2,” and “CD103,” respectively. (A) FITC-specific ear swelling 1 day after elicitation is shown in µm. (B) Relative quantity of mRNA for *Ifng, Il4, Il17a, Il5, Il13* and *Foxp3* was measured by the quantitative real-time PCR in LN cells collected from recipient mice 1 day after elicitation. The values are shown as the mean ± SD from three independent measurements, and the levels of significance for the differences are: *** for p<0.005 and n.s. for not significant. (C) Results of flow cytometry analysis of intracellular cytokines in CD4^+^ T cells from LNs collected from recipient mice 1 day after elicitation are shown. The numbers in the panels indicate the percentages of IFN-γ^+^IL-4^–^CD4^+^ T cells, IFN-γ^–^IL-4^+^CD4^+^ T cells, IFN-γ^+^IL-17A^–^CD4^+^ T cells, and IFN-γ^–^IL-17A^+^CD4^+^ T cells in the total CD4^+^ T cells. (D) The percentages of IFN-γ^+^IL-4^–^CD4^+^ T cells, IFN-γ^–^IL4^+^CD4^+^ cells, and IFN-γ^–^IL-17A^+^CD4^+^ T cells in total CD4^+^ T cells are illustrated in bar graphs. Values in the Y-axis indicate the mean ± SD from three separate experiments, * indicates that the difference is statistically significant (p<0.05) and n.s. indicates that the difference is not statistically significant. (E) FITC-specific IgG1 and IgG2a in the sera of mice that received a transfer of FITC^+^MGL2^+^ cells or FITC^+^CD103^+^ cells were determined. The y-axis indicates the relative antibody binding to FITC. Mean ± SD from 9 separate experiments are shown, and *, ***, and n.s. indicate p<0.05, p<0.005, and difference not statistically significant, respectively. (A–E) The experiments were independently performed more than three times.

The results shown above demonstrated that the levels of cytokines and other molecules involved in Th1-promotion or cross-presentation were lower in MGL2^+^ dDCs compared to CD103^+^ dDCs. MGL2^+^ dDCs produced very low levels of IL-12, which is known to be a key cytokine for Th1 differentiation [43], [44]. Additionally, we previously demonstrated that MGL2^+^ dDCs could induce CHS sensitization *in vivo* [34], indicating that MGL2^+^ dDCs can induce an immune response *in vivo* in CHS. Therefore, we assessed the type of immune response induced by the transfer of MGL2^+^ dDCs *in vivo*. FITC^+^MGL2^+^ dDCs and FITC^+^CD103^+^ dDCs were isolated from skin-draining LNs 1 day after sensitization for CHS and subcutaneously injected into naïve mice to compare the role of these dDC subsets in the induction of different types of immune responses. Ear swelling was elicited by FITC to a similar extent in mice that received transfers of these two subsets (**Fig. 5A**). Therefore, both FITC^+^MGL2^+^ dDCs and FITC^+^CD103^+^ dDCs were capable of CHS sensitization. The expression levels of *Il4* in LN cells were much higher in mice that received FITC^+^MGL2^+^ dDCs than those in mice that received FITC^+^CD103^+^ dDCs or those in untreated mice (**Fig. 5B**). After stimulation with PMA and ionomycin in the presence of brefeldin A, the percentage of IL-4^+^CD4^+^ T cells in the total CD4^+^ T cells of the draining LNs was significantly higher in mice that received FITC^+^MGL2^+^ dDCs than in mice that received FITC^+^CD103^+^ dDCs or in untreated mice (**Figs. 5C and 5D**). In addition, the FITC-specific IgG1 response was significantly higher in mice that received FITC^+^MGL2^+^ dDCs than in mice that received FITC^+^CD103^+^ dDCs or in untreated mice (**Fig. 5E**). These results strongly suggest that MGL2^+^ dDCs, but not CD103^+^ dDCs, skew the immune response toward a Th2-type *in vivo* in CHS. Interestingly, the expression levels of *Il5* and *Il13* in LN cells were not different among these mice (**Fig. 5B**), suggesting that MGL2^+^ dDCs are specialized to induce *Il4* in CD4^+^ T cells. After transfer of FITC^+^MGL2^+^ dDCs or FITC^+^CD103^+^ dDCs, the expression levels of *Ifng, Il17a* and *Foxp3* in LN cells in the recipient mice were not significantly different from those in non-sensitized mice (**Fig. 5B**). The percentage of IL-17A^+^CD4^+^ T cells in the total CD4^+^ T cells from the LNs of these mice was not significantly different from that in the total CD4^+^ T cells of draining LNs from untreated mice (**Figs. 5C and 5D**). The percentage of IFN-γ^+^CD4^+^ T cells in the total CD4^+^ T cells from the draining LNs of mice that received FITC^+^CD103^+^ dDCs was slightly higher than that in mice that received FITC^+^MGL2^+^ dDCs or untreated mice, but the difference was not statistically significant (**Figs. 5C and 5D**). The FITC-specific IgG2a response was not significantly different but was slightly higher in mice that received FITC^+^CD103^+^ dDCs compared to mice that received FITC^+^MGL2^+^ dDCs or untreated mice (**Fig. 5E**). From these results, it is likely that CD103^+^ dDCs comprise a DC subset that has the potential to skew the immune response toward a Th1-type *in vivo* during the sensitization phase of CHS, as previously suggested [69].

### MGL2^+^ dDCs alone cannot induce antigen-specific IL-4 production from T cells *in vitro*

Although a significant contribution of MGL2^+^ dDCs to the Th2-type immune response is clear, we thought it was important to determine whether MGL2^+^ dDCs alone induce antigen-specific IL-4 production from T cells. FITC^+^MGL2^+^ dDCs and FITC^+^CD103^+^ dDCs were isolated from skin-draining LNs 1 day after sensitization for CHS and co-cultured with CD4^+^ T cells isolated from DO11.10*Rag2* ^–/–^ mice in the presence of OVA_323–339_ peptide. The percentage of cytokine-producing T cells 3 or 5 days after co-cultivation and cytokine concentrations in the culture supernatant 3 or 5 days after co-cultivation were measured. In the absence of OVA_323–339_ peptide or dDCs, T cell proliferation was not observed (data not shown). The percentage of IL-4^+^CD4^+^ T cells co-cultured with FITC^+^MGL2^+^ dDCs was similar to that co-cultured with FITC^+^CD103^+^ dDCs (**Fig. S2A**). The IL-4 concentration in the supernatant of T cells co-cultured with FITC^+^MGL2^+^ dDCs was similar to that of T cells co-cultured with FITC^+^CD103^+^ dDCs (**Fig. S2B**). In addition, the percentage of IFN-γ^+^CD4^+^ T cells and the IFN-γ concentration in the supernatant of T cells co-cultured with FITC^+^CD103^+^ dDCs were similar to those after co-culture with FITC^+^MGL2^+^ dDCs (**Figs. S2A and S2B**). These results indicated that MGL2^+^ dDCs and CD103^+^ dDCs alone could not skew the immune response toward Th2 and Th1, respectively, *in vitro*.

### Th2-type humoral response is induced by targeting MGL2^+^ dDCs *in vivo*

**Figure 6:**
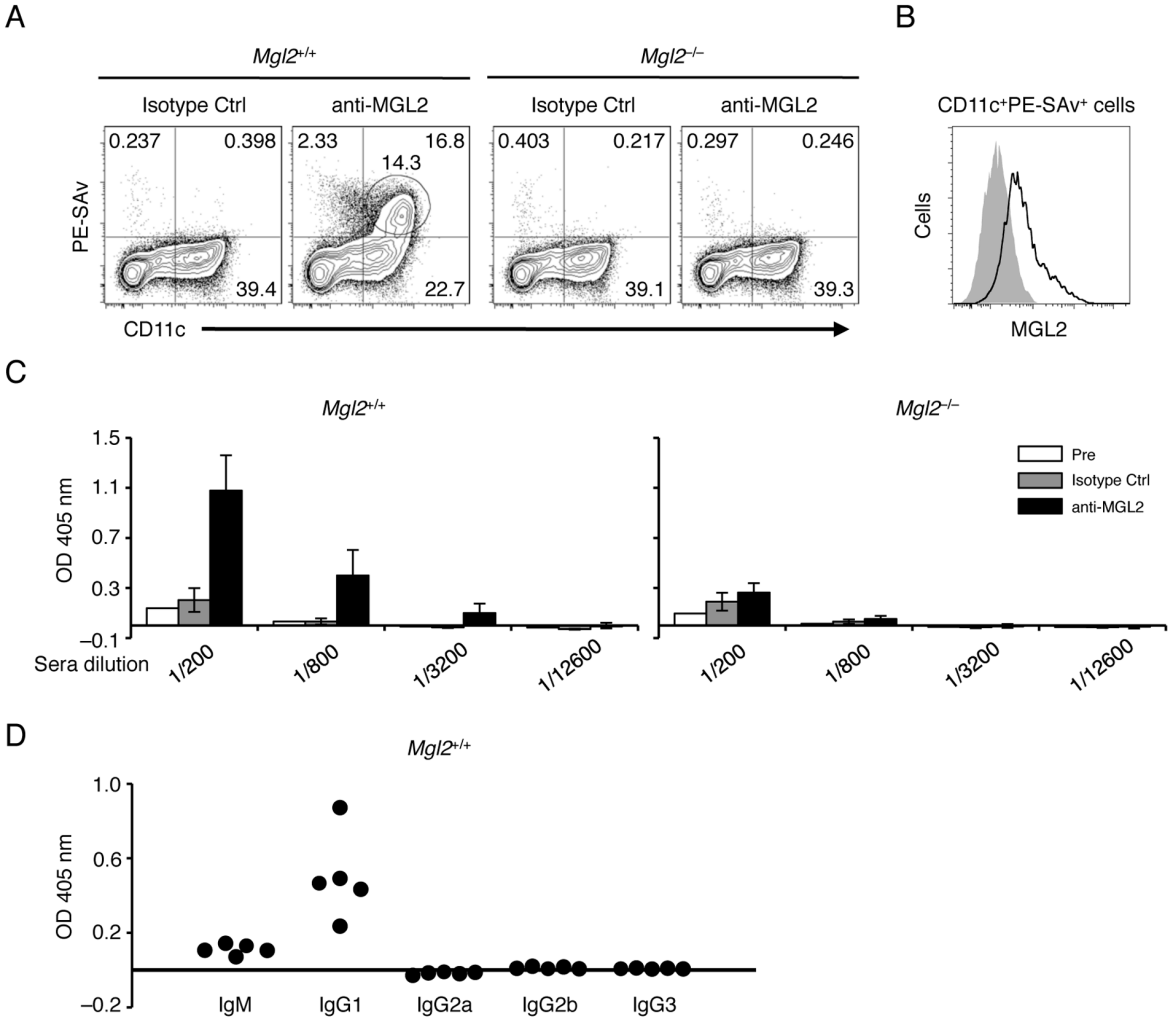
A Th2-type humoral response is induced by targeting MGL2^+^ dDCs *in vivo*. (A) One day after the injection of biotinylated anti-MGL2 mAbs or rat IgG2a (isotype control) into *Mgl2* ^+/+^ or *Mgl2* ^–/–^ mice, cells from draining LNs were subjected to flow cytometry analysis for biotin residues internalized into CD11c^+^ DCs using PE-SAv. (B) Flow cytometry analysis of surface MGL2 on the cells, gated for the levels of PE-SAv binding and CD11c circled in the panel A. (C) Antibodies specific for rat IgG2a in sera were detected 1 week after the injection of rat anti-MGL2 mAbs or rat IgG2a (isotype control) into *Mgl2* ^+/+^ mice or *Mgl2* ^–/–^ mice. (D) Sera obtained 1 week after the injection of anti-MGL2 mAbs into *Mgl2* ^+/+^ mice were assessed for antibody isotypes that were specific for rat IgG2a. (A–D) The experiments were independently performed three times.

We tested *in vivo* whether MGL2^+^ dDCs skewed the immune response toward a Th2-type when an antigen is targeted to MGL2. Molecules reactive to MGL2 were previously shown to be efficiently internalized, processed, and presented to CD4^+^ T cells, but the type of the immune response was not known [33]. Therefore, we immunized mice with a rat anti-MGL2 mAb together with LPS as an adjuvant, and subsequent immune responses to rat IgG were examined. Anti-MGL2 mAbs, but not isotype controls, were internalized by MGL2^+^ dDCs in *Mgl2* ^+/+^ mice. Similar uptake did not occur in equivalent cells from *Mgl2* ^–/–^ mice (**Fig. 6A**), indicating that anti-MGL2 mAbs were internalized through MGL2. An antibody response specific for rat IgG was observed in the immunized *Mgl2* ^+/+^ mice but not in *Mgl2* ^+/+^ mice immunized with isotype controls or in *Mgl2* ^–/–^ mice immunized with rat anti-MGL2 mAbs or with isotype controls (**Fig. 6B**). IgG1 was the main detected subclass in *Mgl2* ^+/+^ mice immunized with anti-MGL2 mAbs (**Fig. 6C**). These results indicate that a Th2-type humoral response took place against an antigen that was taken up by MGL2^+^ dDCs *in vivo*. When LPS was not supplemented, the rat IgG-specific antibody response did not occur (data not shown). Therefore, maturation of MGL2^+^ dDCs, which was induced by LPS but not by cross-linking of cell surface MGL2, seems to be necessary for an efficient Th2-type humoral response.

## Discussion

The type of an immune response is likely to depend upon the subsets and the degree of maturation of the antigen presenting cells that are present. In the skin immune system, LCs and CD103^+^ dDCs were previously reported to promote Th17-type and Th1-type immune responses, respectively [16], [17]. However, the types of immune response promoted or suppressed by other dDC subsets *in vivo* had yet to be elucidated [17]. In the present study, we show that MGL2 serves as a marker for a dDC subset distinct from CD103^+^ dDCs or LCs both under naïve and sensitized conditions in a model to elicit CHS. Using MGL2 as a marker, we isolated and characterized MGL2^+^ dDCs from skin and skin-draining lymph nodes.

The increase in the expression levels of co-stimulatory molecules after sensitization was similar among MGL2^+^ dDCs, DN DCs, and CD103^+^ dDCs. Thus, the capacities of these skin-derived DC subsets for T cell activation [70], [71] are likely to be comparable to each other *in vivo*. These results were consistent with previous work in other laboratories showing that both LCs and CD103^+^ dDCs are involved in CHS sensitization [12], [25], [26], [27], [28] and suggest that MGL2^+^ dDCs also play important roles for sensitization leading to CHS *in vivo* [34].

By encyclopedic transcriptome analyses, expression levels of Th1-promoting cytokines, especially *Il12b*, and molecules known to be involved in cross-presentation, especially *Xcr1, Tlr3* and *Clec9a*, were lower in MGL2^+^ dDCs than in CD103^+^ dDCs and the differences in mRNA levels were verified by the quantitative real-time PCR. In contrast, MGL2^+^ dDCs contained almost similar levels of transcripts for Th2-promoting molecules compared to CD103^+^ dDCs. In addition, MGL2^+^ dDCs have been shown to produce low levels of IL-12, which was previously known to skew the immune response toward a Th1-type [1], [72], even after sensitization for CHS. MGL2^+^ dDCs were also shown to produce lower levels of IL-6, which skewed the immune response toward a Th17-type [73] after sensitization for CHS (data not shown). These results lead us to hypothesize that MGL2^+^ dDCs and CD103^+^ dDCs initiate different types of immune responses.

To prove or disprove this hypothesis, we isolated MGL2^+^ dDCs and CD103^+^ dDCs from mice sensitized with FITC and transferred them to naïve mice to test the type of immune response initiated by each subset. MGL2^+^ dDCs were shown to transfer antigen-specific Th2-type immunity, as revealed by the induction of IL-4^+^CD4^+^ T cells and a FITC-specific IgG1 response *in vivo.* In contrast, CD103^+^ dDCs were likely to render a Th1-type immune response, based on our finding that these cells expressed *Il12b* at high levels, produced high amount of IL-12_p40_ and IL-12_p70_ *in vitro*, and induced IFN-ã^+^CD4^+^ T cells and FITC-specific IgG2a *in vivo*. Takeshita and co-workers reported that neutralization of IL-4 during the sensitization phase resulted in a reduction of CHS by more than 60% [74], demonstrating that IL-4 played a significant role in the initiation of CHS. It is likely that MGL2^+^ dDCs initiate CHS through the regulation of IL-4 production by T cells.

We demonstrated that MGL2^+^ dDCs and CD103^+^ dDCs alone could not skew the immune response toward Th2 and Th1, respectively, *in vitro*. These results suggest the following two possibilities. First, FITC^+^MGL2^+^ dDCs alone cannot skew the immune response toward a Th2-type in an antigen-dependent manner unless additional help comes from other cells, such as basophils, previously shown to contribute to the Th2-type immune response [75], [76], and FITC^+^CD103^+^ dDCs are in a similar situation for the Th1 response in the context of this model. Secondly, an *in vivo* niche may play an important additional role in skewing for a Th2 or Th1 response. MGL2^+^ dDCs did not induce *Il5* and *Il13* expression in LN cells *in vivo* after sensitization for CHS, suggesting that MGL2^+^ dDCs represent a subset specialized to induce IL-4 production from CD4^+^ T cells.

The numbers of the transcripts that are known to promote a Th2-type immune response, including *Il10* [63], *Tnfsf4* [64], [65], *jag1* [66], [67], *jag2* [66], and *Crlf2* (TSLP receptor) [68], in MGL2^+^ dDCs were not largely different from those in CD103^+^ dDCs, both under naïve and sensitized conditions in our transcriptome analysis. Therefore, in the Th2-type immune response initiated by MGL2^+^ dDCs *in vivo*, these molecules might not directly be involved. Very low numbers of transcripts were found for molecules involved in the Th1-type immune response or in cross-presentation, such as *Il12b, Xcr1, Tlr3* and *Clec9a* [43], [44], [48], [53], [54], [55], [56], [57], [58], [59], [60], [61], in MGL2^+^ dDCs. At the protein level, IL-12_p40_ and IL-12_p70_ levels were very low, both under naïve and sensitized conditions, and IL-6 was down-regulated under sensitized conditions in MGL2^+^ dDCs (data not shown). The expression of co-stimulatory molecules that are important for T cell activation [70], [71] was elevated to a similar extent as other skin-derived DCs after sensitization for CHS. These results suggest that MGL2^+^ dDCs stimulated naïve T cells via co-stimulatory molecules but failed to induce T cell differentiation toward a Th1-type or Th17-type response. Consequently, the immune response was likely to skew toward a Th2-type *in vivo* in CHS. MacDonald reported that DCs might induce a Th2-type immune response in the absence of IL-12, and our interpretation is consistent with their findings [77]. Guilliams and co-workers reported that CD103^–^ dDCs, corresponding mostly to MGL2^+^ dDCs, induced antigen-specific Tregs *in vitro* under a naïve state [78]. In the present study, however, MGL2^+^ dDCs did not induce *Foxp3* expression in LN cells *in vivo* in CHS. The discrepancy was most likely due to the increased expression of co-stimulatory molecules after sensitization for CHS.

In the present study, we did not extensively investigate the characteristics of DN DCs, which should correspond mostly to LCs. According to our analyses, DN DCs were shown to produce low levels of IL-12_p40_ under naïve and sensitized conditions, and the expression levels of co-stimulatory molecules on DN DCs, as shown by flow cytometry analysis, were almost the same as MGL2^+^ dDCs and CD103^+^ dDCs. However, we previously demonstrated that MGL2^+^ dDCs migrate to the outer areas of the T cell cortex in skin-draining LNs 1 day after FITC-painting, whereas the other groups demonstrated that LCs migrate into the T cell cortex 4 days after FITC- or TRITC-painting [24], [34], [79], [80]. Judging from these results, MGL2^+^ dDCs and LCs might act in distinct areas and at distinct time points despite the fact that IL-12_p40_ expression is similar between MGL2^+^ dDCs and LCs. Therefore, MGL2^+^ dDCs and LCs may play different roles in the course of the immune response in CHS.

A rat monoclonal antibody specific for MGL2 was used to target MGL2^+^ DCs to induce an immune response *in vivo* [33]. A Th2-type humoral response was induced, as shown by the antibody subclass, which was consistent with the role of MGL2^+^ dDCs in CHS. In addition, encyclopedic transcriptome analysis and the quantitative real-time PCR analysis revealed that the expression profiles of C-type lectin receptors in MGL2^+^ dDCs and CD103^+^ dDCs were distinct from each other. MGL2^+^ dDCs expressed *Clec4n* at high levels, whereas CD103^+^ dDCs expressed *Clec9a* at high levels. It was previously shown that cross-presentation and a Th1-type immune response were induced when Langerin, DEC205, or Clec9A were targeted [57]. These results collectively indicate that carbohydrate moieties of proteins direct antigens to defined subsets of DCs, which incorporate, process and present antigens to lead to a subsequent immune response, and suggest that the exogenous antigen taken up into MGL2^+^ dDCs through MGL2 induces Th2-type immune responses.

In conclusion, we demonstrated that dDCs displaying MGL2 on their surfaces were phenotypically and functionally distinct from LCs or CD103^+^ dDCs, and MGL2^+^ dDCs induced a Th2-type immune response. A causal relationship between the surface expression of MGL2 and the Th2-type immune response induced by MGL2^+^ dDCs remains to be determined.

## Supporting Information

Figure S1 **The expression of co-stimulatory molecules on dDC subsets in skin-draining LNs.** Flow cytometry analysis for the expression of co-stimulatory molecules on MGL2^+^ dDCs, MGL2^–^CD103^–^ skin-derived DCs, and CD103^+^ dDCs from skin-draining LNs under a naïve state or 1 day after FITC painting. MGL2^+^ dDCs are shown as “MGL2,” MGL2^–^CD103^–^ skin-derived DCs are shown as “DN,” and CD103^+^ dDCs are shown as “CD103.” (A) MHCII^high^MGL2^+^ dDCs (MGL2), MHCII^high^ MGL2^–^CD103^–^ skin-derived DCs (DN), and MHCII^high^CD103^+^ dDCs (CD103) in skin-draining LNs from mice under a naïve state were analyzed for the expression of the indicated co-stimulatory molecules. The number indicates the MFI of each co-stimulatory molecule on each skin-derived DC subset. (B) FITC^+^MGL2^+^ dDCs (MGL2), FITC^+^MGL2^–^CD103^–^ skin-derived DCs (DN), and FITC^+^CD103^+^ dDCs (CD103) in skin-draining LNs from mice 1 day after FITC painting were analyzed for the expression of the indicated co-stimulatory molecules. The number indicates the MFI of each co-stimulatory molecule on each skin-derived DC subset. (A–B) The experiments were independently performed three times.
(TIF)

Figure S2 **Induction of cytokines in CD4^+^ T cells by co-culture with MGL2^+^ dDCs or CD103^+^ dDCs** ***in vitro*** **.** (A) FITC^+^MGL2^+^ dDCs or FITC^+^CD103^+^ dDCs were co-cultured with CD4^+^ T cells from DO11.10*rag2* ^–/–^ mice *in vitro*. Three days later, these cells were stimulated with PMA/ionomycin in the presence of brefeldin A, and intracellular cytokine levels of T cells were determined by flow cytometry. In these panels, T cells co-cultured with FITC^+^MGL2^+^ dDCs are shown as “MGL2,” and T cells co-cultured with FITC^+^CD103^+^ dDCs are shown as “CD103.” The numbers indicate the percentages of IFN-γ^+^IL-4^–^CD4^+^ T cells and IFN-γ^–^IL-4^+^CD4^+^ T cells in total CD4^+^ T cells. (B) Concentrations of IFN-γ and IL-4 in culture supernatants were determined before stimulation with PMA/ionomycin. Culture supernatants in the presence of FITC^+^MGL2^+^ dDCs are shown as “MGL2,” and culture supernatants in the presence of FITC^+^CD103^+^ dDCs are shown as “CD103.” (A–B) The experiments were independently performed three times.
(TIF)

Table S1 Encyclopedic transcriptome analysis of MGL2^+^ dDCs and CD103^+^ dDCs. The number of transcripts in MHCII^high^MGL2^+^ cells from naïve mice, MHCII^high^CD103^+^ cells from naïve mice, FITC^+^MGL2^+^ cells from mice 1 day after FITC painting, and FITC^+^CD103^+^ cells from mice 1 day after FITC painting are shown. Nine categories – cytokines, chemokines, TNF ligand superfamily members, cytokine receptors, chemokine receptors, TNF receptor superfamily members, C-type lectins, TLRs, and NLRs – were chosen. The lists include items whose expression levels were greater than 15 in MGL2^+^ dDCs, CD103^+^ dDCs, or both, either under a naïve state or 1 day after FITC painting. MHCII^high^MGL2^+^ dDCs are shown as “Naïve MGL2,” MHCII^high^CD103^+^ dDCs are shown as “Naïve CD103,” FITC^+^MGL2^+^ dDCs are shown as “Sensitized MGL2” and FITC^+^CD103^+^ dDCs are shown as “Sensitized CD103.”
(TIF)

Table S2 The expression of Th2-promoting molecules on MGL2^+^ dDCs and CD103^+^ dDCs. Comparison of transcripts of Th2-promoting molecules chosen from the encyclopedic transcriptome analysis of MHCII^high^MGL2^+^ cells from naïve mice, MHCII^high^CD103^+^ cells from naïve mice, FITC^+^MGL2^+^ cells from mice 1 day after FITC painting, and FITC^+^CD103^+^ cells from mice 1 day after FITC painting. MHCII^high^MGL2^+^ dDCs are shown as “Naïve MGL2,” MHCII^high^CD103^+^ dDCs are shown as “Naïve CD103,” FITC^+^MGL2^+^ dDCs are shown as “Sensitized MGL2,” and FITC^+^CD103^+^ dDCs are shown as “Sensitized CD103.” The relative numbers of each transcript are indicated.
(TIF)

Table S3 The list of primers used in the quantitative real-time PCR.
(TIF)
