# Correction: A Unique Dermal Dendritic Cell Subset That Skews the Immune Response toward Th2

**DOI:** 10.1371/journal.pone.0093236

**Published:** 2014-03-18

**Authors:** The PLOS ONE Staff

The alpha symbol (α) appears incorrectly throughout the manuscript. All cases of "CD8a’" should appear as "CD8α".

The beta symbol (β) appears incorrectly in the “Antibodies” section of the Materials and Methods. The correct name is "TCRVβ (JR2)" and appears incorrectly as “TCRVa∧8 (JR2)”.

The gamma symbol (γ) appears incorrectly in the fourth paragraph of the discussion. The correct name is "IFN-γ^+^CD4^+^ T cells" and appears incorrectly as “IFN-a ^∼+^CD4^+^ T cells”.

Figure 3E is incorrect. The authors have provided a corrected version here.

**Figure 3:**
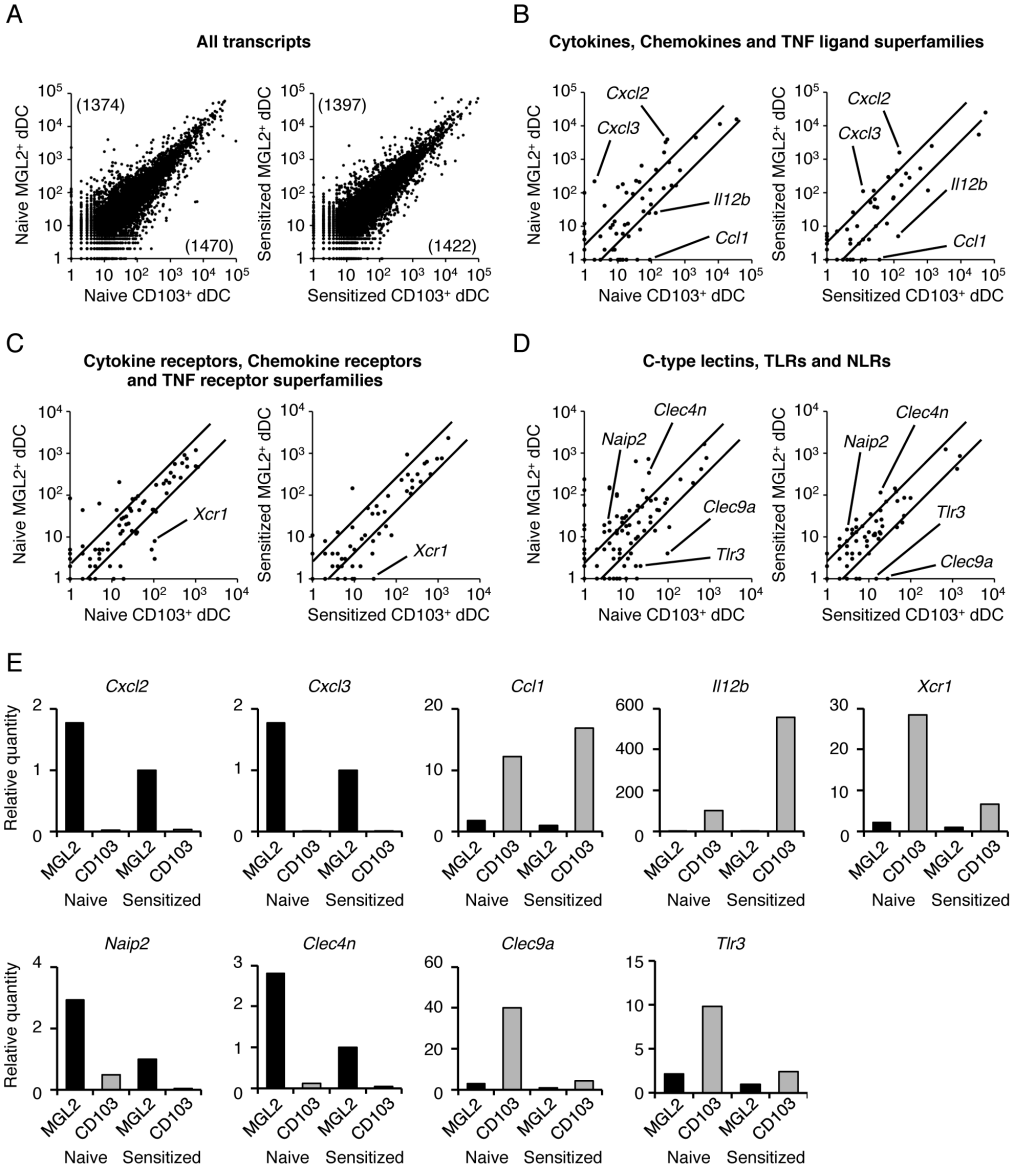
Encyclopedic transcriptome analysis of MGL2^+^ dDCs and CD103^+^ dDCs and results of quantitative real-time PCR for the transcripts suggested by the transcriptome analysis to have differential levels. (A–D) Reciprocal two-dimensional plots of the results of transcriptome analysis among MHCII^high^MGL2^+^ cells, MHCII^high^CD103^+^ cells, FITC^+^MGL2^+^ cells and FITC^+^CD103^+^cells. In these panels, MHCII^high^MGL2^+^ dDCs are indicated as “Naïve MGL2^+^ dDC,” MHCII^high^CD103^+^ dDCs are indicated as “Naïve CD103^+^ dDC,” FITC^+^MGL2^+^ dDCs are indicated as “Sensitized MGL2^+^ dDC,” and FITC^+^CD103^+^ dDCs are indicated as “Sensitized CD103^+^ dDC.” (A) All transcripts of cells from naïve mice and cells from sensitized mice are shown. The numbers in the parentheses indicate the number of transcripts expressed 3 times greater than the other dDC subset. (B–D) The numbers of transcripts in selected categories are plotted. In the comparisons, the names of the transcripts are indicated when they fit the following criteria: (1) the number is greater than 15 in MGL2^+^ dDCs or in CD103^+^ dDCs, and (2) the difference in the number is 5-fold or greater both before and after sensitization. The diagonal lines represent the border for 3-fold differences. (B) Transcripts of cytokines, chemokines and TNF ligand superfamily members are shown. (C) Transcripts of cytokine receptors, chemokine receptors, and TNF receptor superfamily members are shown. (D) Transcripts of C-type lectins, TLRs and NLRs are shown. (E) The quantitative real-time PCR analysis of the expression of indicated genes (*Cxcl2, Cxcl3, Ccl1, Il12b, Xcr1, Naip2, Clec4n, Clec9a* and *Tlr3*). They were chosen from the categories indicated above (B), (C), and (D) and the differences between MGL2^+^ dDCs and CD103^+^ dDCs based on the transcriptome analysis, appeared to be significant under both untreated and sensitized conditions (Figs. 3B–D). (A–E) Transcriptome analysis was performed once. The quantitative real-time PCR analysis was independently performed more than two times.
