# Supplementary figures and images for: A Unique Dermal Dendritic Cell Subset That Skews the Immune Response toward Th2
Source: PLoS One. 2013 Sep 9;8(9):e73270. doi: 10.1371/journal.pone.0073270 (PMC3767795; doi:10.1371/journal.pone.0073270)

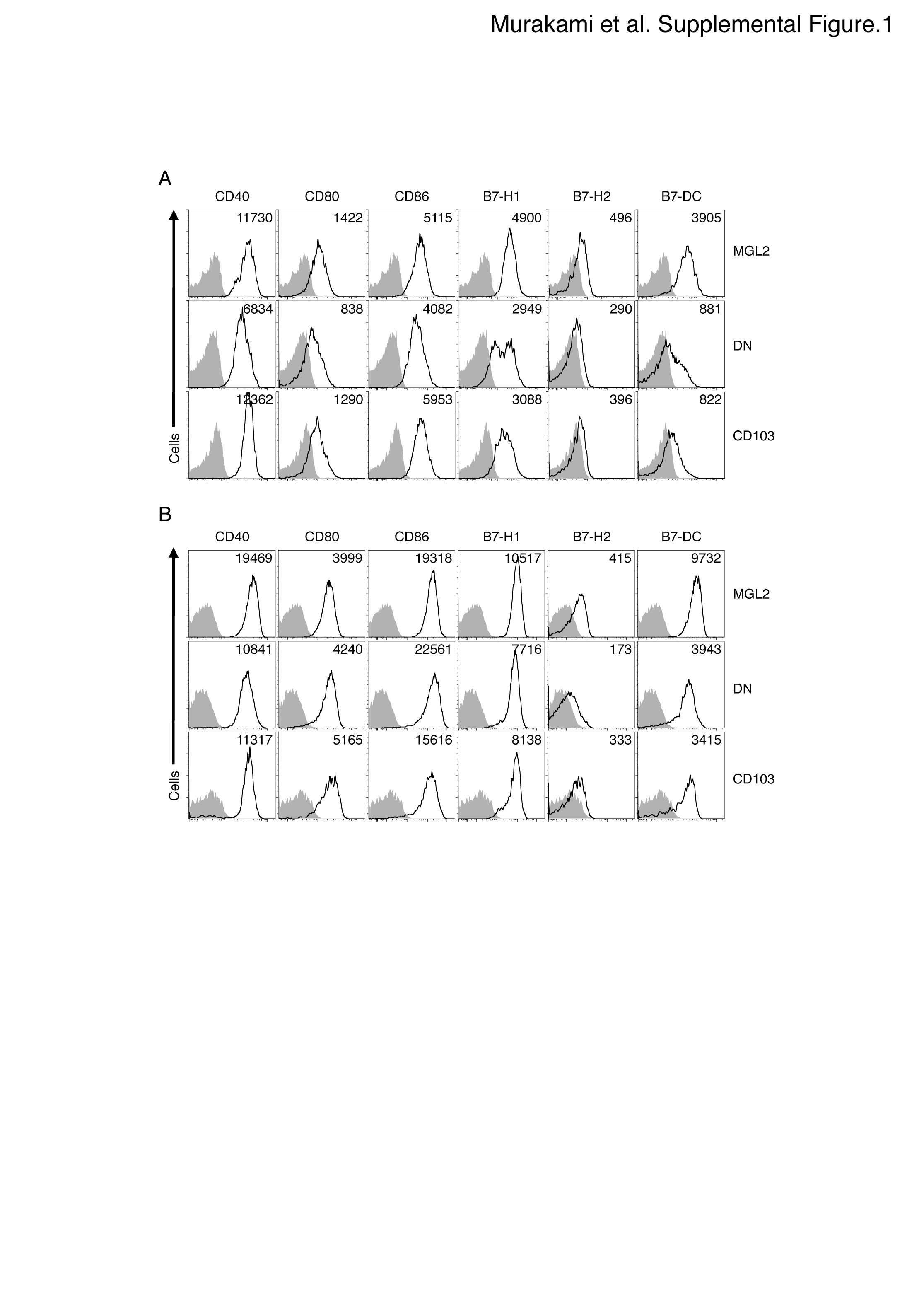

Supplement: Figure S1 — The expression of co-stimulatory molecules on dDC subsets in skin-draining LNs. Flow cytometry analysis for the expression of co-stimulatory molecules on MGL2+ dDCs, MGL2–CD103– skin-derived DCs, and CD103+ dDCs from skin-draining LNs under a naïve state or 1 day after FITC painting. MGL2+ dDCs are shown as “MGL2,” MGL2–CD103– skin-derived DCs are shown as “DN,” and CD103+ dDCs are shown as “CD103.” (A) MHCIIhighMGL2+ dDCs (MGL2), MHCIIhigh MGL2–CD103– skin-derived DCs (DN), and MHCIIhighCD103+ dDCs (CD103) in skin-draining LNs from mice under a naïve state were analyzed for the expression of the indicated co-stimulatory molecules. The number indicates the MFI of each co-stimulatory molecule on each skin-derived DC subset. (B) FITC+MGL2+ dDCs (MGL2), FITC+MGL2–CD103– skin-derived DCs (DN), and FITC+CD103+ dDCs (CD103) in skin-draining LNs from mice 1 day after FITC painting were analyzed for the expression of the indicated co-stimulatory molecules. The number indicates the MFI of each co-stimulatory molecule on each skin-derived DC subset. (A–B) The experiments were independently performed three times. (TIF) [file pone.0073270.s001.tif]

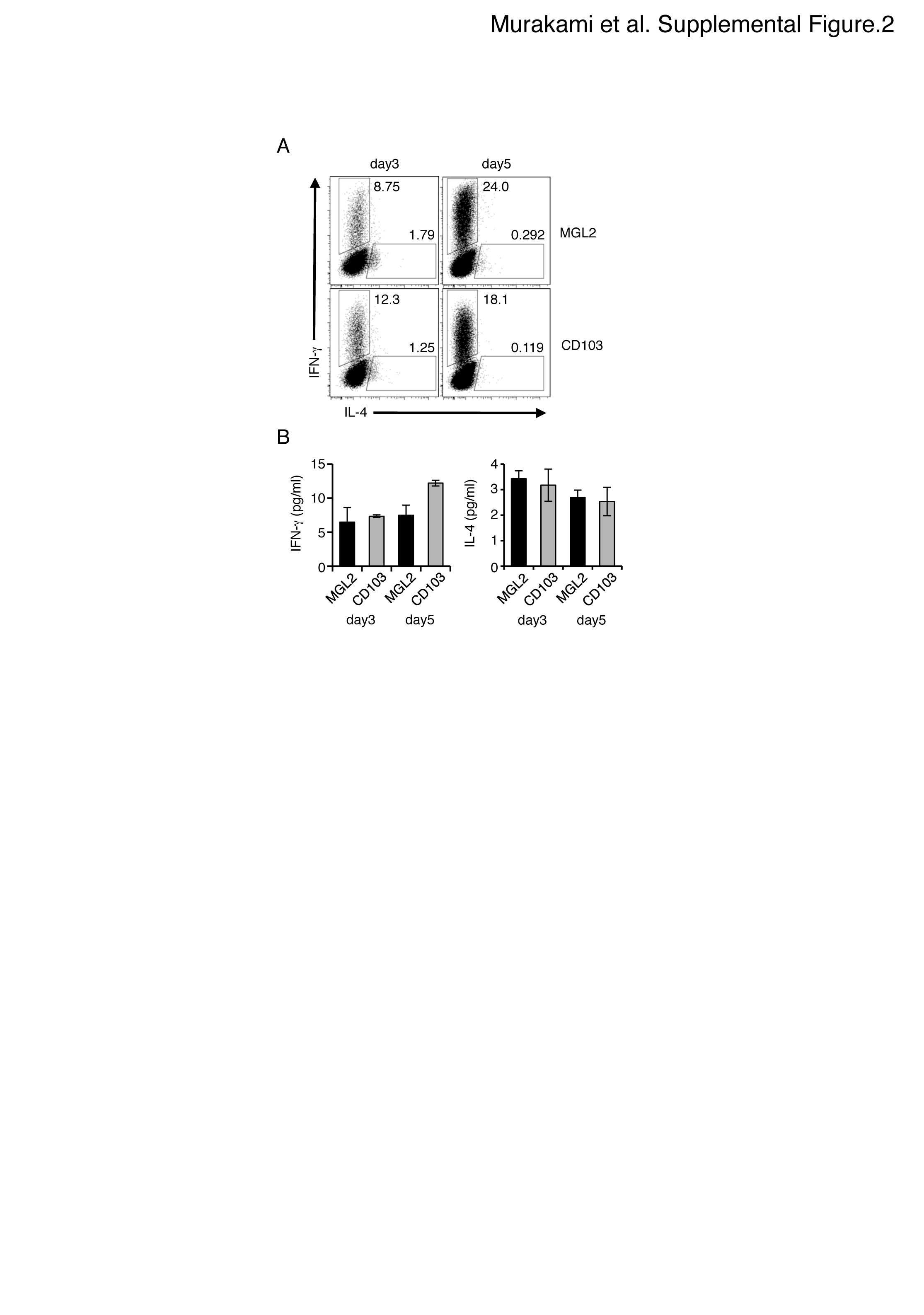

Supplement: Figure S2 — Induction of cytokines in CD4+ T cells by co-culture with MGL2+ dDCs or CD103+ dDCs in vitro . (A) FITC+MGL2+ dDCs or FITC+CD103+ dDCs were co-cultured with CD4+ T cells from DO11.10rag2 –/– mice in vitro. Three days later, these cells were stimulated with PMA/ionomycin in the presence of brefeldin A, and intracellular cytokine levels of T cells were determined by flow cytometry. In these panels, T cells co-cultured with FITC+MGL2+ dDCs are shown as “MGL2,” and T cells co-cultured with FITC+CD103+ dDCs are shown as “CD103.” The numbers indicate the percentages of IFN-γ+IL-4–CD4+ T cells and IFN-γ–IL-4+CD4+ T cells in total CD4+ T cells. (B) Concentrations of IFN-γ and IL-4 in culture supernatants were determined before stimulation with PMA/ionomycin. Culture supernatants in the presence of FITC+MGL2+ dDCs are shown as “MGL2,” and culture supernatants in the presence of FITC+CD103+ dDCs are shown as “CD103.” (A–B) The experiments were independently performed three times. (TIF) [file pone.0073270.s002.tif]

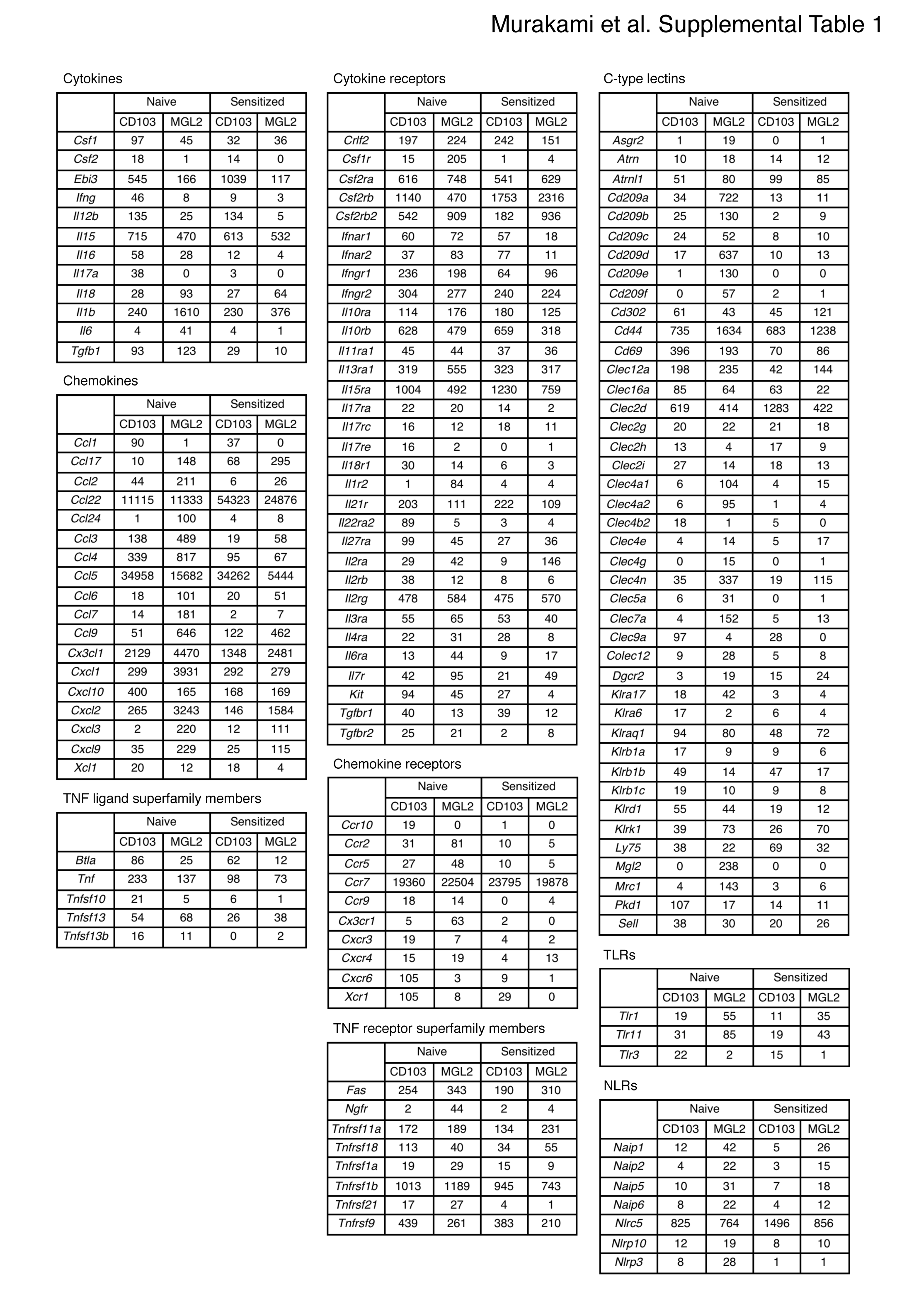

Supplement: Table S1 — Encyclopedic transcriptome analysis of MGL2+ dDCs and CD103+ dDCs. The number of transcripts in MHCIIhighMGL2+ cells from naïve mice, MHCIIhighCD103+ cells from naïve mice, FITC+MGL2+ cells from mice 1 day after FITC painting, and FITC+CD103+ cells from mice 1 day after FITC painting are shown. Nine categories – cytokines, chemokines, TNF ligand superfamily members, cytokine receptors, chemokine receptors, TNF receptor superfamily members, C-type lectins, TLRs, and NLRs – were chosen. The lists include items whose expression levels were greater than 15 in MGL2+ dDCs, CD103+ dDCs, or both, either under a naïve state or 1 day after FITC painting. MHCIIhighMGL2+ dDCs are shown as “Naïve MGL2,” MHCIIhighCD103+ dDCs are shown as “Naïve CD103,” FITC+MGL2+ dDCs are shown as “Sensitized MGL2” and FITC+CD103+ dDCs are shown as “Sensitized CD103.” (TIF) [file pone.0073270.s003.tif]

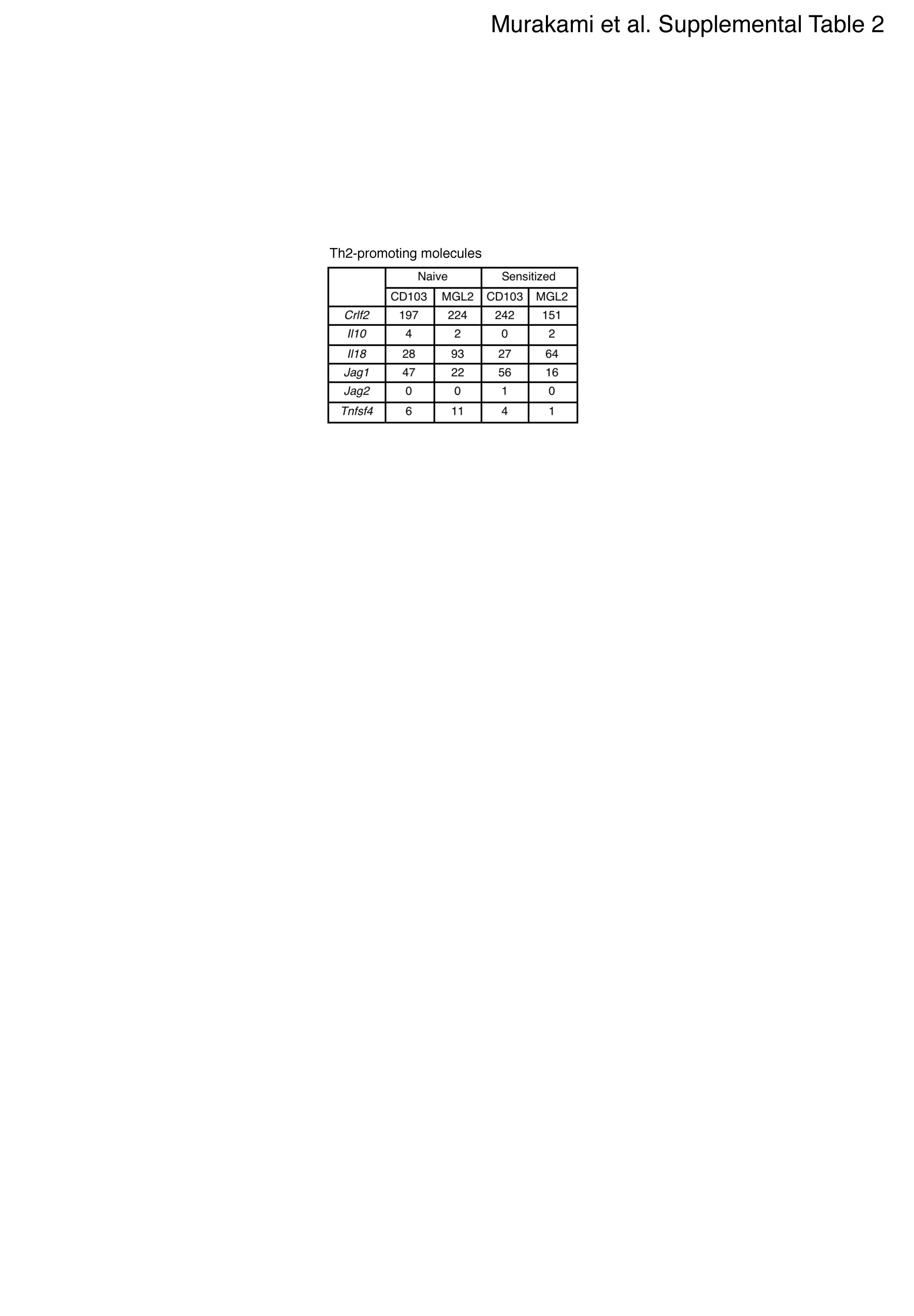

Supplement: Table S2 — The expression of Th2-promoting molecules on MGL2+ dDCs and CD103+ dDCs. Comparison of transcripts of Th2-promoting molecules chosen from the encyclopedic transcriptome analysis of MHCIIhighMGL2+ cells from naïve mice, MHCIIhighCD103+ cells from naïve mice, FITC+MGL2+ cells from mice 1 day after FITC painting, and FITC+CD103+ cells from mice 1 day after FITC painting. MHCIIhighMGL2+ dDCs are shown as “Naïve MGL2,” MHCIIhighCD103+ dDCs are shown as “Naïve CD103,” FITC+MGL2+ dDCs are shown as “Sensitized MGL2,” and FITC+CD103+ dDCs are shown as “Sensitized CD103.” The relative numbers of each transcript are indicated. (TIF) [file pone.0073270.s004.tif]

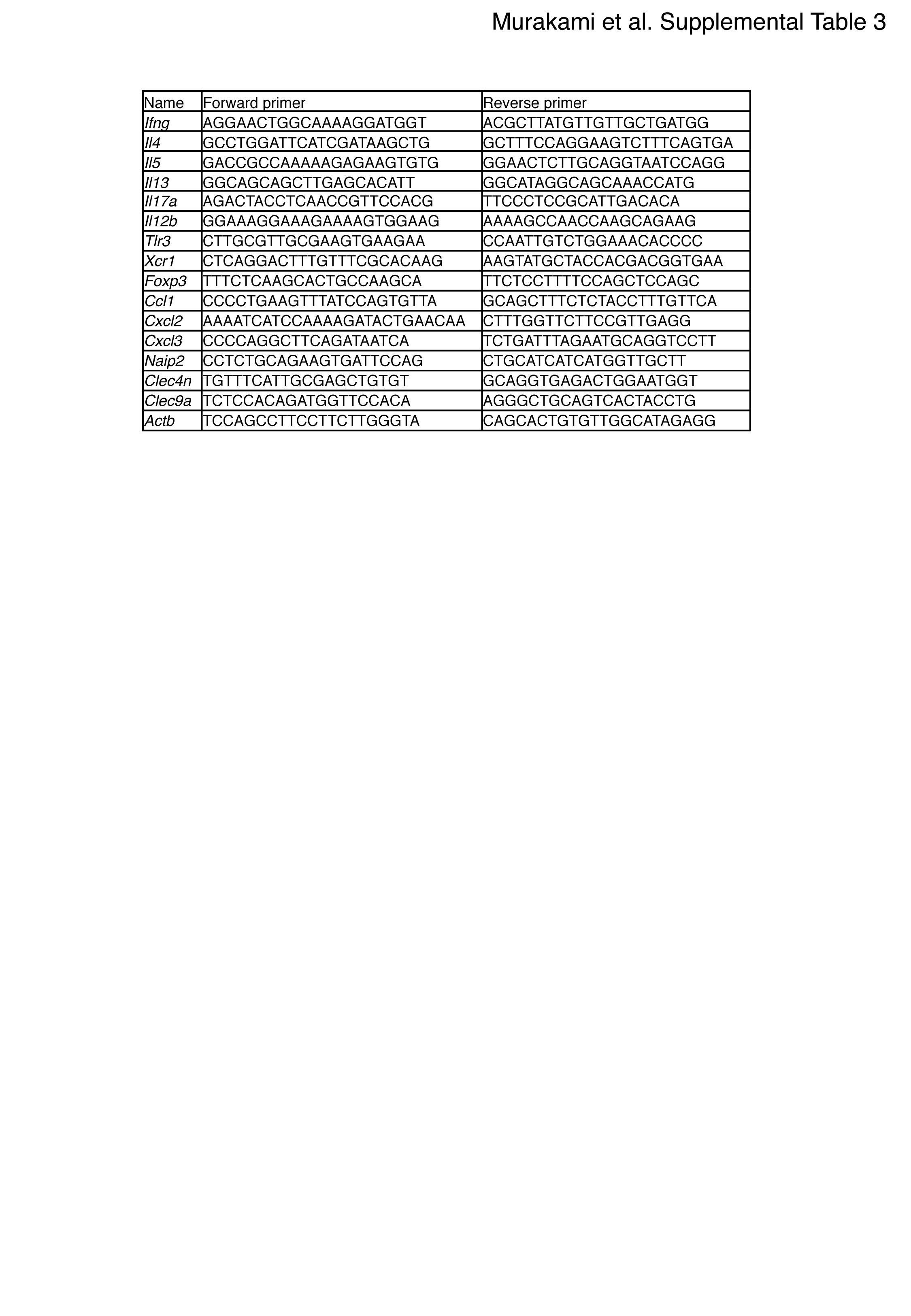

Supplement: Table S3 — The list of primers used in the quantitative real-time PCR. (TIF) [file pone.0073270.s005.tif]
